# Stand structure adjustment influences the biomass allocation in naturally generated *Pinus massoniana* seedlings through environmental factors

**DOI:** 10.3389/fpls.2022.997795

**Published:** 2022-10-28

**Authors:** Wenchun He, Yu Wang, Xiao Wang, Xiaochen Wen, Tianyi Li, Mengting Ye, Gang Chen, Kuangji Zhao, Guirong Hou, Xianwei Li, Chuan Fan

**Affiliations:** Sichuan Province Key Laboratory of Ecological Forestry Engineering on the Upper Reaches of the Yangtze River, State Forestry and Grassland Administration Key Laboratory of Forest Resources Conservation and Ecological Safety on the Upper Reaches of the Yangtze River, College of Forestry, Sichuan Agricultural University, Chengdu, China

**Keywords:** biomass allocation, environmental factors, natural regeneration, *Pinus massoniana*, stand spatial structure characteristic indexes, structural equation models

## Abstract

The natural regeneration of seedlings is a key factor for forest succession. Nevertheless, studies explaining the mechanism of growth and biomass allocation in regenerated seedlings after disturbance are lacking. Therefore, we measured the growth, biomass accumulation, and biomass allocation in current-age seedlings of *Pinus massoniana* after selective logging (logging of competitive trees, *LCT*; logging of inferior trees, *LIT*; and unlogged control, *CK*), and established structural equation models (SEMs) among the spatial structure characteristic indexes of the stand, environmental factors, and biomass allocation in different organs. As compared to the *CK*, the mingling index (*M*), uniform angle index (*W*), opening degree (*O*), soil organic carbon (SOC), available nitrogen (SAN), available phosphorus (SAP), available potassium (SAK), and bulk density (SBD) significantly increased (*p* < 0.05), while the competition index (*CI*) and neighborhood comparison (*U*) significantly decreased after logging (*p* < 0.05). After the *LCT*, seedling branch biomass improved, with an increase in the ground-diameter, crown-root ratio, and seedling quality index. More biomass was allocated to foliage and roots by an increase in the height and height-diameter ratio under the *LIT*. In the *CK*, increasing stem biomass helped the seedlings absorb and utilize more light. The Pearson correlation coefficient showed that biomass allocation to organs was independent, and seedlings adopted the strategies of heterogeneous adaptation and growth, thereby resulting in the separation of the allocation patterns among the organs. As per the redundancy analysis (RDA), *CI* was the main factor in biomass allocation. Environmental factors had direct effects on biomass allocation to organs, while the stand spatial structure characteristic indexes had indirect effects on biomass allocation based on SEMs. In summary, the *LCT* had significant, albeit indirect, effects on SOC, SAN, and SBD by reducing the *CI* for the regeneration and growth of seedlings in the stand, which was of great significance to the sustainable development of the forest stand of *P. massoniana*.

## Highlights

- Biomass allocation of naturally regenerated seedlings have changed after logging.
- The competition index was the main factor to biomass allocation of organs.
- The stand spatial structure characteristic indexes had indirect effects on the biomass allocation based on SEMs through environmental factors.
- Compared to logging of inferior trees, logging of the competitive trees was more conducive to the regeneration and growth of seedlings.

## Introduction

The natural regeneration of trees, *via* their reproductive capacity to form seedlings and saplings that eventually reach the state of uneven forests, is an important pathway for the self-recovery of species and the stability of the forest ecosystem (Lai et al., 2019). Previous studies on regeneration focused more on the settlement, density, composition, and distribution patterns. For example, de Carvalho et al. (2017) found that the types, numbers, and distribution patterns of naturally regenerated seedlings increased after selective logging, which was in line with the report of Zhao et al. (2021). By contrast, Rivett et al. (2016) and Kitenberga et al. (2020) found that logging may reduce the abundance and renewal rate of naturally regenerated seedlings. Nevertheless, although there is a consensus on the settlement, composition, and distribution of seedlings, an understanding of the growth and photosynthate allocation among organs (such as branch, foliage, stem, and root) in naturally regenerating seedlings is lacking.

Photosynthate allocation has a very important position in the growth strategies of plants, as well as substance-recycling and energy-flow in ecosystems (Yan et al., 2016; Song et al., 2021; Umaña et al., 2021). However, the measurement processes for photosynthesis during the production, transfer, and consumption stages are difficult due to the complexity of the engineering and technology required (McCarthy and Enquist, 2007; Umaña et al., 2021). For instance, Bebre et al. (2021) found that excessive drought can reduce the allocated biomass in the plant's aboveground parts. Plants are also reported to take a series of survival strategies under low-light conditions, including absorption of photosynthetically active radiation and a decrease in carbon consumption by reducing underground biomass allocation and root-shoot ratio, along with an increase in plant height, specific leaf area, and canopy area (Sevillano et al., 2016). Recently, Song et al. (2021) concluded that biomass allocation is influenced by both plant ontogeny and leaf traits. Therefore, biomass allocation at the organ level (branch, foliage, stem, and root) is often used, instead of photosynthate allocation, to gain a deeper understanding of the strategy and adaptability involved in overall survival in heterogeneous environments (Song et al., 2021; Umaña et al., 2021).

To date, biomass allocation is predominantly explained using environmental factors. For instance, Poorter et al. (2012) concluded that environmental factors, such as ozone and CO_2_, affect biomass allocation among the different organs of plants to achieve balanced growth and development. Fay et al. (2015) asserted that the availability of nutrients (nitrogen, phosphorus, and potassium) may increase belowground biomass allocation rather than aboveground biomass allocation to improve net primary production in the terrestrial ecosystem. Bebre et al. (2021) emphasized that plants may employ a trade-off among organs by changing stem biomass to adapt to the light intensity. Undoubtedly, changes in environmental factors are caused by disturbances in the structure of the forest stand and the overall forest ecosystem (Wang et al., 2022b). Since environmental factors can affect biomass allocation among plant organs (Poorter et al., 2012), the spatial structure will also affect it. Thus, whether and how the spatial structure of a forest directly or indirectly affects the growth and biomass allocation during natural regeneration remains unclear (Tavankar et al., 2022).

Previous studies have focused on the correlation between environmental factors and growth rather than the causal relationships among the different elements of the forest ecosystems. Mounting evidence has shown that the structural equation model (SEM) has many advantages in directly or indirectly exploring such hypothetical relationships because of its flexible modeling form. For instance, Grace et al. (2016) used the SEM to reveal mechanisms that link productivity and plant species richness using within-site variations in soil suitability and shading. The work by Angelini et al. (2017) concluded that biomass allocation was indirectly impacted by soil organic carbon, where the clay percentage was a direct factor dictating the cation exchange capacity of the soil. Using the SEMs, Qi et al. (2021) showed that species-dimension biomass inequality indirectly increased with productivity *via* enhanced soil water content and with species diversity *via* increased precipitation. Cheng et al. (2021) used the SEMs to discover the effects of rainfall, year, and their interactions that could directly affect the aboveground biomass and also indirectly affect the leaf economics spectrum. Thus, the SEM can be used to better explore the direct and indirect pathways and positive or negative effects of biomass allocation, spatial structure, and/or environmental factors.

*Pinus massoniana* is among the main species used for vegetation restoration and wood utilization due to its strong adaptability, rapid growth rate, and high yield in China (Wang et al., 2022b). Nonetheless, *P. massoniana* may suffer problems in the regeneration of its seedlings due to unreasonable logging and extensive managements with low nutrient inputs in large areas (Wang et al., 2022b). To explore the growth and biomass allocation in naturally regenerated seedlings and understand the direct or indirect role of factors pertaining to stand structure in biomass allocation, we measured tree growth, biomass accumulation, and biomass allocation in current-age seedlings after selective logging and established SEMs among the stand structure factors, environmental factors, and biomass allocation in seedlings. We provide relevant data and a theoretical basis for the sustainable development of forest stands.

## Materials and methods

### Description of study sites

**Figure 1:**
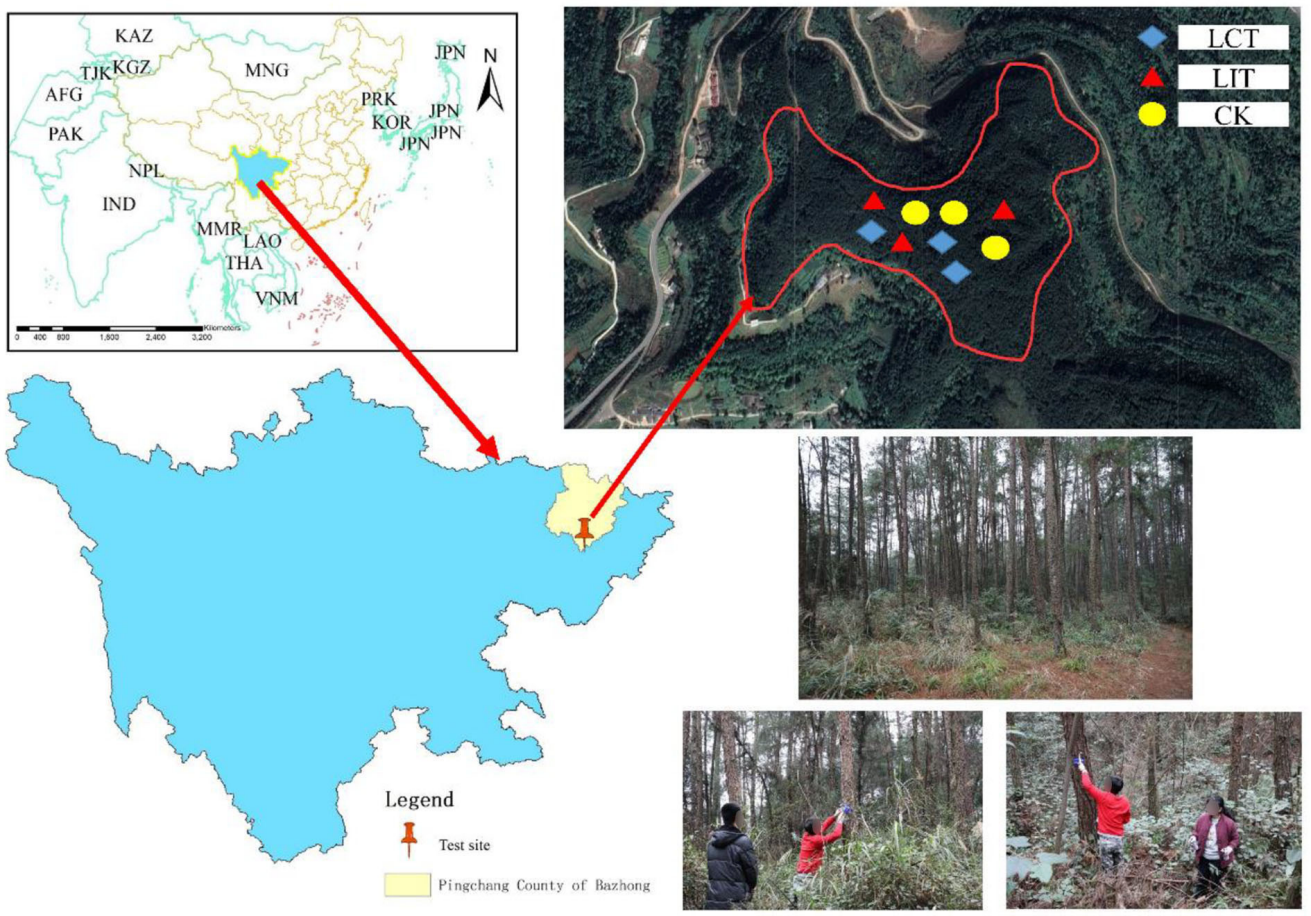
The map of the study sites (Location map of Liumen Town, Pingchang County, Bazhong City, Sichuan Province, China). The logging methods are *LCT*, logging of competitive trees; *LIT*, logging of inferior trees; *CK*, unlogged control.

The study site is located in Damaoping, Liumen Town, Pingchang County, Sichuan Province, China (107°1′34″-107°4′40″E, 31°31′37″-31°36′53″N, Figure 1). The average altitude and slope of the site were 720 m and 15°, respectively, making it a stepped ridge-valley landform. It belongs to the subtropical humid monsoon climate zone with an annual average temperature of 16.8°C, average annual sunshine of 1,365.5 h, average relative humidity of 79%, a frost-free period of 298 days, and average annual rainfall of 1,138.2 mm (the data were obtained from January 2017 to December 2021, and the distance from the meteorological station to the study area was about 25 km).

Before selective logging, the total area of the *P. massoniana* near-mature forest (forest age = 20 years) in Damaoping was 50.0 hm^2^; the average forest density was about 2,500 plants·hm^−2^, and the average diameter at breast height (DBH) was about 17.21 cm. The soil was yellow and rich in aluminum and silicon, and the soil pH was 5.0–7.0. The soil layer was shallow and ranged from 25 to 40 cm. There were a few arbor species present, including *Cupressus funebris, Toona sinensis, Alnus Cremastogyne Burk*, and *Broussonetia papyrifera*. The understory vegetation was mainly *Coriaria nepalensis, Camellia japonica, Myrsine Africana, Smilax china, Rhododendron simsii, Dicranopteris dichotoma, Psilotum nudum*, and *Osmunda japonica*.

### Experimental design

In this study, the tree density of *P. massoniana* for crop-management purposes was set to 200–400 plants·hm^−2^ based on a previous experiment. In October 2017, with the initial afforestation density of 2,500 plants·hm^−2^ and the preserved density later recorded to be 1,800 plants·hm^−2^, logging tree density was set to 300 plants·hm^−2^ using logging of competitive trees (*LCT*); logging of inferior trees was established with the same intensity (*LIT*) and unlogged forest was established as the control (*CK*). The area of each plot was 20 × 30 m. A total of 9 plots (3 treatments × 3 replicates) were established (Table 1). To reduce the interference across treatments, a buffer zone of approximately 20 m was established among the plots by digging a trench. Before logging, we investigated the number of trees in the plot, and used an optical theodolite (BB17-TDJ6E, BJ Haifuda Co., Beijing, China) and a handheld laser rangefinder (Leica, BJ Xinlanyasi Co., Beijing, China) to measure the azimuth angle and horizontal distance of each tree, respectively, based on the inspection rule (the DBH and tree height) for each tree (DBH ≥ 5 cm or the height of the tree ≥ 80 cm).

**Table 1:** The basic information of sample plots.

**Indexes**		**Unlogged control (*CK*)**	**Logging of competitive trees (*LCT*)**	**Logging of inferior trees (*LIT*)**
Elevation (m)		720–723	720–723	720–723
Slope position		Top	Top	Top
Slope		15°	15°	15°
Before logging	Density (plants·hm^−2^)	1,533 ± 51a	1,488 ± 47a	1,429 ± 43a
	Average DBH (cm)	17.13 ± 0.23a	17.23 ± 0.17a	17.18 ± 0.33a
After logging	Density (plants·hm^−2^)	1,533 ± 51a	1,205 ± 47b	1,000 ± 43c
	Average DBH (cm)	17.13 ± 0.23b	16.43 ± 0.32b	18.81 ± 0.53a
Current	Density (plants·hm^−2^)	1,533 ± 51a	1,205 ± 47b	1,000 ± 43c
	Average DBH (cm)	19.37 ± 0.61b	24.27 ± 0.57a	20.51 ± 0.74b

Data shown are the mean ± standard deviation (n = 3). Different lowercase letters indicated significant differences among the treatments (*p* < 0.05).

*LCT*: The Kraft' tree classification method was used to classify the forest trees. First, according to the status and potential of the trees, we classified each tree in the plot into dominant trees (defined as trees with the greatest tree heights or DBH forming a large canopy above the main forest layer that receive the best light conditions), sub-dominant trees (compared to the dominant trees, a sub-dominant tree is smaller in height, DBH, and crown size, but it forms a nearly uniform and symmetrical crown in the main forest layer), moderate trees (compared with dominant or sub-dominant trees, a moderate tree has smaller tree height and DBH with a narrower canopy that reaches the main forest layer, but the canopy is compressed in at least one direction), pressed trees (defined as a tree that grows very slowly in height and has a small DBH with a narrow and compressed canopy, which majorly reaches below the main canopy), and dying or dead trees (these are below the canopy and cannot receive normal light; their growth is weak causing them to be close to dying or even dead due to external causes, such as disease, insect infestation, or aging. These trees may have withered naturally but may not have yet fallen). Second, based on a previous forest classification, we categorized each tree as the crop-management tree (dominant trees in the forest stands constituted crop-management trees, which presented strong vitality, vigorous growth, straight stem shape, good quality, and no damage), ecological crop-management trees (endangered tree species, famous and old-age trees, and mixed tree species for maintaining the structure of the forest and preserving biodiversity), competitive trees (trees which were adjacent to crop-management trees and same tree species that adversely affect the growth of crop-management trees, such as the canopy area that squeezed more than one-third of the crop-management tree), and general trees (excluding the above three types). We also marked the trees with red, yellow, white, and blue paints to indicate their categories, i.e., crop management, ecological crop-management, competitive, and general trees, respectively. According to the density and intensity of the management, we selected and marked the crop-management trees to obtain a maximally uniform distribution in the plots. Finally, we cut the competitive trees. Considering the reserving and harvesting intensities, when the management plan was prepared, we calculated the initial number of harvested plants in the plot, and appropriately adjusted the number of competitive trees to prevent insufficient or over-intensified harvesting. The logged trees were kept in their native place as forest litter. No management was performed after logging.

*LIT*: first, based on the tree height, crown area, and orientation, the Kraft' tree classification method was used to classify the forest trees as mentioned above (dominant trees, sub-dominant trees, moderate trees, pressed trees, and dying or dead trees were marked as I-tree, II-tree, III-tree, IV-tree, and V-tree, respectively). Second, we also used red, yellow, white, blue, and green paint for marking the categories, respectively. Finally, based on logging of the competitive trees of the same intensity, we cut from the V-tree, IV-tree, and parts of the III-tree. The same management strategy as used for the *LCT* was applied after logging.

### Determination of the spatial structure characteristic index

After selective logging, we determined the spatial structure characteristic indexes of the retention trees in the forest. First, we determined the height and DBH with a measuring ruler and vernier caliper (DBH ≥ 5 cm, or tree height ≥ 80 cm). The position (the azimuth angle and horizontal distance) was measured with an optical theodolite and handheld laser rangefinder for each tree. Second, the information on the position was transformed to the relative coordinate position (x_1_, y_1_), (x_2_, y_2_)... (x_n_, y_n_) for each tree through the slope correction with Origin 9.0 software (OriginLab Inc., Northampton, MA, USA). Third, we calculated the mingling index (*M*, Equation 1) (Hui et al., 2018), competition index (*CI*, Equation 2) (Hui et al., 2018), neighborhood comparison (*U*, Equation 3) (Hui et al., 2018), uniform angle index (*W*, Equation 4) (Hui et al., 2018), and opening degree (*O*, Equation 5) (Gadow et al., 2012) using the following equations (Table 2):

**Table 2:** Equations for calculating the spatial structure characteristic indexes.

**Number**	**Index**	**Equation**	**Where**	**References**
1	Mingling index	$Mi=\frac{1}{n}\overset{n}{\underset{j=1}{\sum }}Vij$	$V_{ij}=\{\frac{1}{0},\frac{speciesi\neq speciesj}{otherwise}$	Hui et al., 2018
2	Competition index	$CIi=\overset{n}{\underset{j=1}{\sum }}\frac{Dj}{D\text{i}Lij}$		Hui et al., 2018
3	Neighborhood comparison	$U\text{i}=\frac{1}{n}\overset{n}{\underset{j=1}{\sum }}Kij$	$K_{ij}=\{\frac{1}{0},\frac{D_{i}<D_{j}}{otherwise}$	Hui et al., 2018
4	Uniform angle index	$Wi=\frac{1}{n}\overset{n}{\underset{j=1}{\sum }}Zij$	${Zi}_{j}=\{\frac{1}{0},\frac{\alpha_{ij}<\alpha_{0}}{otherwise}$	Hui et al., 2018
5	Opening degree	$K\text{i}=\frac{1}{n}\overset{n}{\underset{j=1}{\sum }}\frac{Lij}{Hij}$		Gadow et al., 2012

Where, n is the number of neighbors, and in this study, *n* = 4; *D*_*i*_ is the diameter at breast height (DBH) of the *i*^*th*^ reference tree; *D*_*j*_ is the DBH of the *j*^*th*^ neighboring tree; *L*_*ij*_ is the distance between the *i*^*th*^ reference tree and the *j*^*th*^ neighboring tree; *H*_*ij*_ is the height between the reference tree and four neighboring trees; α_*ij*_ is the horizontal angle between the reference tree and four neighboring trees; a standard angle α_0_ =72°. All the structure-based indices have five possible values: 0.00, 0.25, 0.50, 0.75, and 1.00.

### Sampling and determination of the growth and biomass allocation

#### Measuring the number, plant height, and ground-diameter of seedlings

In accordance with the number of branches (that is, the *P. massoniana* will form a one-turn branch each year in the subtropical region), plant height, and the position of the previous survey data and photos, we accurately determined the age of the naturally regenerated seedlings of *P. massoniana*. In January 2021, we counted the number of the 3-year-old seedlings in the plot and measured their heights with a wooden ruler (accuracy: 0.01 cm) and ground-diameter with a vernier caliper (accuracy: 0.01 mm) (Supplementary Table 1).

#### Calculating the number of sampling plants

Previous studies on the natural regeneration of seedlings adopted the method of directly harvesting seedlings. However, harvesting too many seedlings can destroy the sustainable management of a forest, while harvesting too few plants introduces inaccuracy in the results. To minimize the negative influence on the plot and the precision of later experiments, we calculated the number of plants that needed to be sampled through the test-efficiency equation (Equation 6). Due to the large differences in the plant heights, we chose the ground-diameter as the measurement standard and set the inspection efficiency to 80%. The “pwr” R package in the R 4.1.2 platform (R Foundation for Statistical Computing, Vienna, AT) was used. Significance (α), inspection efficiency (1-β), and effect value (*f*) were set to 0.05, 80%, and 0.5532677, respectively. The ground-diameters under *LCT, LIT*, and *CK* were 4.63, 3.92, and 3.08 mm, respectively, and the overall mean value was 3.64 mm. According to Equation (6), we found that at least 12 plants had to be sampled from each plot to achieve 80% inspection efficiency.


(6) $$\begin{array}{l} f=\sqrt{\frac{\sum_{i=1}^{k}P_{i}^{*}{\left(\mu_{i}-\mu \right)}^{2}}{\alpha^{2}}} \end{array}$$

Where, *f* was the effect value. P_i_ = n_i_/N, n_i_ is the number of observations in the i group, N is the total number of observations; μ_i_ is the mean value of the i group; μ is the total mean; α^2^ is the variance within the group; k is the number of groups; and n is the sample size in each group.

#### Determination of biomass allocation in organs

We randomly excavated 12–13 3-year-old *P. massoniana* seedlings using the whole-plant harvesting method from each plot. We excavated the root system and associated soil while avoiding any damage to the root system of the plants. After cutting off the main roots and above-ground components using a branch shear, the entire root system was placed in a numbered sealing bag. The roots scattered in the soil were collected and placed in another sealing bag. The excavation process was based on the horizontal and vertical distribution of the root system for positioning. When collecting fine roots, the soil blocks were lightly broken to prevent a large amount of soil from falling off, which otherwise would confuse the classification of fine roots. Each soil block was fully sorted to ensure that a certain number of fine roots was collected from each seedling. *P. massoniana* roots were distinguished from other roots based on their color and smell. Some error was expected in the collection of fine roots (diameter ≤ 2 mm). As the broken fine roots only accounted for a very small part of the biomass (< 1%), they would be of little to no significance in the calculation of biomass allocation. After being brought back to the laboratory, the samples were rinsed with running water and sorted, and according to the branches, foliage, stems, and roots, were packed into envelopes, put at a constant temperature of 105°C for 15 min, and dried at 80°C to achieve a constant weight. They were then transferred to an electronic balance (accuracy of 0.0001 g, AS-213/EJ-123, HN Jingmai Ins., Henan, China) and weighed to obtain the biomass of branches, foliage, stems, and roots. Finally, we calculated the biomass allocation ratio for each part (Table 3, Equations 7–10), crown-root ratio (C/R ratio, Table 3, Equation 11), height-diameter ratio (H/D ratio, Table 3, Equation 12), and seedling quality index (SQI, Table 3, Equation 13).

**Table 3:** Equations for calculating biomass allocation of organs.

**Number**	**Index**	**Equation**
7	Branch mass fraction	$\text{Branch mass fraction (BMF)=}\frac{branch\;biomass}{branch\text{biomass+foliage biomass+stem biomass+root biomass}}$
8	Foliage mass fraction	$Foliage\text{mass fraction (LMF)=}\frac{foliage\text{biomass}}{branch\text{biomass+foliage biomass+stem biomass+root biomass}}$
9	Stem mass fraction	$Stem\text{mass fraction (SMF)=}\frac{stem\text{biomass}}{branch\text{biomass+foliage biomass+stem biomass+root biomass}}$
10	Root mass fraction	$Root\text{mass fraction (RMF)=}\frac{root\text{biomass}}{branch\text{biomass+foliage biomass+stem biomass+root biomass}}$
11	Crown-Root ratio	$Crown-root\text{ratio (C/R ratio)=}\frac{branch\text{biomass + foliage biomass}}{root\text{biomass}}$
12	Height-Diameter	$H\text{ei}ght-diameter\;ratio\text{(H/D ratio) =}\frac{height}{ground\;diameter}$
13	Seedling quality index	$seedling\;quality\;index\text{(SQI)=}\frac{branch\;biomass+foliage\;biomass+stem\;biomass+root\;biomass}{H/D\;ratio+C/R\;ratio}$

### Analysis of physical and chemical properties of sampled soil

As the depth of the seedling root system did not exceed 35 cm, we collected the topsoil from a depth of 0–40 cm. In the rectangular plots, we took each soil sample at 6 and 12 m from the center point along the diagonal to the apex of each plot (four soil samples at each diagonal and one at the center point), totaling 9 soil samples for each plot. After removing the litter and humus layer from the soil surface, we excavated soil samples with a soil drill, separated debris, such as stone and roots, mixed the 9 samples from each plot and put ~2.0 kg soil samples into numbered bags, which were brought back to the laboratory for further analyses, including soil water content (SW), soil pH (pH), soil organic carbon (SOC), soil total nitrogen (STN), soil available nitrogen (SAN), soil total phosphorus (STP), soil available phosphorus (SAP), soil total potassium (SK), and soil available potassium (SAK). Six soil cores were sampled simultaneously *in-situ* at about 25 cm depth away from the soil-sampling position by a soil cutting ring (5 cm in diameter), which were used to determine the soil bulk density (SBD) and soil porosity (SP) by weighing the soil and calculating the mass after soils were oven dried at 105°C to a stable weight (Yin et al., 2022). The SW was determined by the drying method (He et al., 2022), pH by the glass electrode method (the water-to-soil ratio was 1:2.5) (He et al., 2022), SOC by the potassium dichromate oxidation-external heating method (He et al., 2022), STN by the Kjeldahl method (He et al., 2022), SAN by alkaline hydrolysis diffusion method (He et al., 2022), STP by the alkali fusion-molybdenum antimony colorimetric method (He et al., 2022), SAP by sodium bicarbonate extraction-molybdenum-antimony colorimetric method (He et al., 2022), SK by extraction-atomic absorption spectrophotometry determination (He et al., 2022), and SAK by atomic absorption spectrophotometry determination after neutral ammonium acetate extraction (He et al., 2022).

### Statistical analyses

Microsoft Excel 2007 (For windows, Microsoft Co., WA, USA) was used to manage, sort, and calculate the data, and the SPSS 20.0 software (SPSS 20.0 for windows, SPSS Ins., Chicago, IL, USA) was used for statistical analyses.

First, the Shapiro-Wilk test and Levene's test were used to evaluate the normality and homogeneity of the variance for each group of variables, and the Box-Cox method was used to transform the non-normal and uneven variables into normality (such as using In or Log logarithmic conversion) when the normality and homogeneity were not met.

Second, the differences in the number, height, ground-diameter, biomass accumulation, and biomass allocation to organs (such as branch, foliage, stem, and root), stand structure characteristic indexes (such as *M, W, U, CI*, and *O*), and soil physical and chemical properties (such as SOC, STN, SAN, STP, SAP, SK, SAK, SW, pH, SBD, and SP) under different treatments were analyzed by one-way ANOVA using SPSS 20.0 (SPSS 20.0 for windows, SPSS Ins., Chicago, IL, USA). These tests were followed by Duncan's multiple comparison method (*p* < 0.05). GraphPad Prism software (version 8.0.2) was used for the illustration of the figures.

Third, Pearson correlation was used to analyze the relationship between stand spatial structure characteristic indexes (such as *M, W, U, CI*, and *O*), environmental factors (such as SOC, STN, SAN, STP, SAP, SK, SAK, SW, pH, SBD, and SP) and biomass allocation to organs (such as BMF, LMF, SMF, and RMF) in the R 4.1.2 platform (R Foundation for Statistical Computing, Vienna, AT) using the R packages, “psych,” “pheatmap,” and “ggcorrplot.” Due to the large differences in the dimensions and values of the indicators, we normalized all indicators with a 0–1 data normalization method.

Fourth, we analyzed important drivers of stand spatial structure characteristic indexes (such as *M, W, U, CI*, and *O*) or environmental factors (such as SOC, STN, SAN, STP, SAP, SK, SAK, SW, pH, SBD, and SP) on the biomass allocation to organs (such as BMF, LMF, SMF, and RMF) with redundancy analysis (RDA) in Canoco software (version 5.0). To decrease collinearity, variables with the highest variance inflation factor (*vif*) values were removed one by one until the *vifs* of all variables in the RDA were lower than 10.

Finally, the SEM was conducted to evaluate the relative importance of stand spatial structure characteristic indexes and environmental factor in controlling biomass allocation to organs. SEM analysis was conducted in AMOS 21.0 (Amos Development Co., Chicago, IL, USA). To develop the final SEM, based on the previous correlation analysis, we omitted stand structure characteristic indexes (such as *M, W, U, CI*, and *O*) and environmental factors (such as SOC, STN, SAN, STP, SAP, SK, SAK, SW, pH, SBD, and SP) that had no significant correlation with the biomass allocation to the different organs. In addition, in the optimal SEM model, we assumed that stand structure characteristic indexes would significantly affect environmental factors, thereby having a significant impact on the biomass allocation to organs. These data were 0–1 normalized and included in the principal component analysis (PCA) by using the R 4.1.2 platform (R Foundation for Statistical Computing, Vienna, AT) using the R the package, “factoextra.” The first component (PC1) of each explanatory group was used as input in the subsequent SEM analysis. Model evaluation was determined by the chi-square test (χ^2^, *p* > 0.05 for a satisfactory fit), the Goodness Fit Index (GFI, GFI > 0.9 for a satisfactory fit), the Normalized Fit Index (NFI, NFI > 0.9 for a satisfactory fit), the standardized root mean square residual (SRMR, SRMR < 0.05 for a satisfactory fit), and the Akaike information criterion (AIC) were used to select the optimal model.

## Results

### Changes in the spatial structure characteristic indexes and environment factors after logging

**Figure 2:**
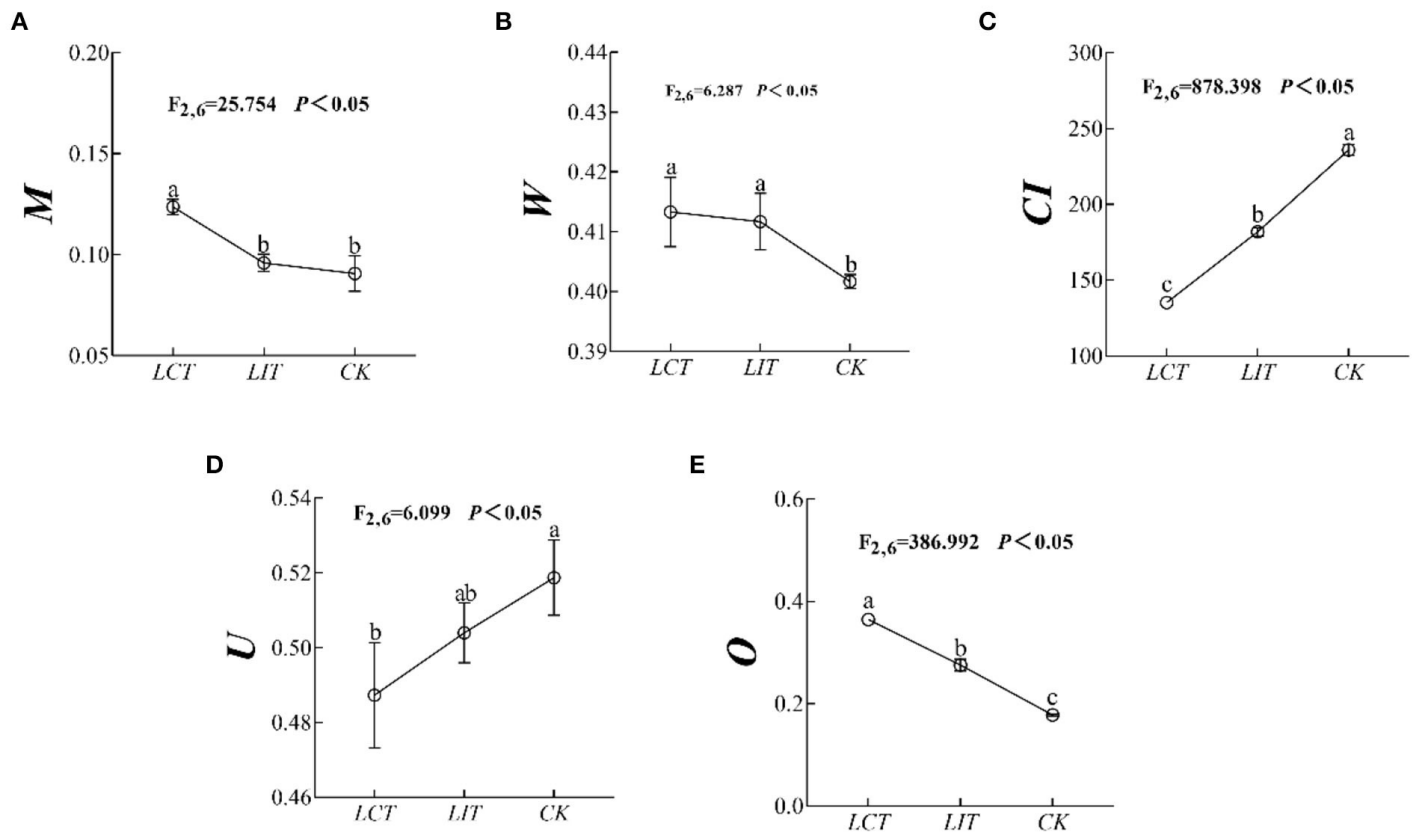
The forest stand spatial structure characteristic index. The logging methods are *LCT*, logging of the competitive trees; *LIT*, logging of the inferior trees; *CK*, unlogged control. The stand spatial structure parameters are *M*, mingling index; *W*, uniform angle index; *CI*, competition index; *U*, neighborhood comparison; and *O*, opening degree. Data shown are the mean ± standard deviation (*n* = 3). Different lowercase letters indicated significant differences among the treatments (*p* < 0.05) in **(A–E)**.

The mingling index (*M*), uniform angle index (*W*), competition index (*CI*), neighborhood comparison (*U*), and opening degree (*O*) of the forests were significantly influenced by the logging treatment (*p* < 0.05, Figure 2, Supplementary Table 2). Specifically, the *M* (*F*_2,6_ = 25.754, *p* < 0.05, Figure 2A), *W* (*F*_2,6_ = 6.287, *p* < 0.05, Figure 2B), and *O* (*F*_2,6_ = 386.992, *p* < 0.05, Figure 2E) were significantly increased (*LCT* > *LIT* > *CK*), while the *CI* (*F*_2,6_ = 878.398, *p* < 0.05, Figure 2C) and *U* (*F*_2,6_ = 6.099, *p* < 0.05, Figure 2D) decreased significantly (*LCT*<*LIT*<*CK*). In other words, *LCT* was beneficial to reducing the competition within forest stands and enlarging the openness of the canopy.

**Figure 3:**
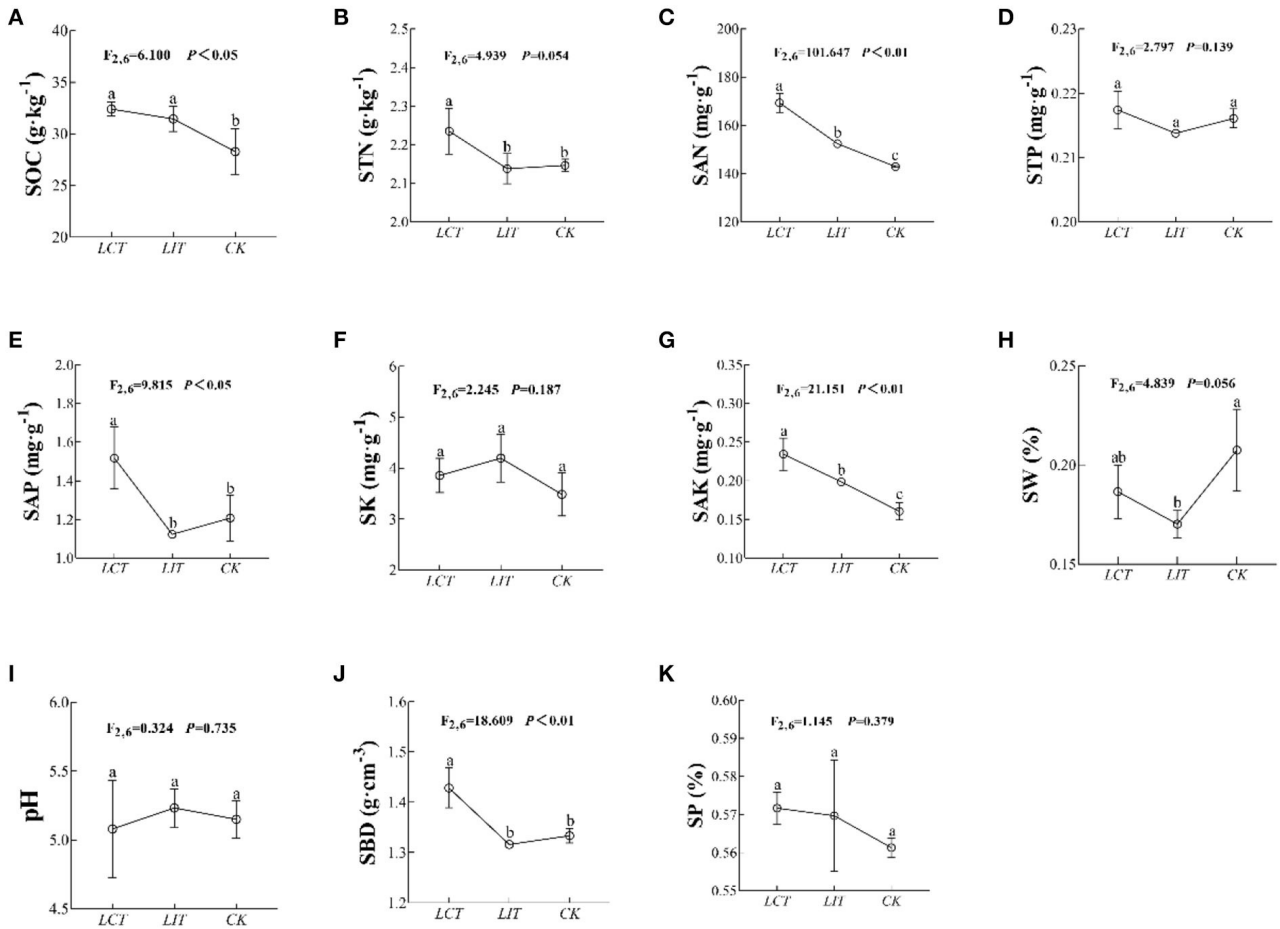
The environment factors under the three treatments. The logging methods are *LCT*, logging of the competitive trees; *LIT*, logging of the inferior trees; *CK*, unlogged control. The soil parameters are SOC, soil organic carbon; STN, soil total nitrogen; SAN, soil alkaline nitrogen; STP, soil total phosphorus; SAP, soil available phosphorus; SK, soil total potassium; SAK, soil available potassium; SW, soil water content; pH, soil pH; SBD, soil bulk density; SP, soil porosity. Data shown are the mean ± standard deviation (*n* = 3). Different lowercase letters indicated significant differences among the treatments (*p* < 0.05) in **(A–K)**.

In addition, the soil organic carbon (SOC), total nitrogen (STN), available nitrogen (SAN), available phosphorus (SAP), available potassium (SAK), and bulk density (SBD) were significantly influenced by the logging treatment (*p* < 0.05, Figure 3, Supplementary Table 3). In both *LCT* and *LIT*, the STN (*F*_2,6_ = 4.939, *p* = 0.054, Figure 3B), STP (*F*_2,6_ = 2.797, *p* = 0.139, Figure 3D), SK (*F*_2,6_ = 2.245, *p* = 0.187, Figure 3F), SW (*F*_2,6_ = 4.839, *p* = 0.056, Figure 3H), pH (*F*_2,6_ = 0.324, *p* = 0.735, Figure 3I), and SP (*F*_2,6_ = 1.145, *p* = 0.379, Figure 3K) were not significantly influenced (*p* > 0.05). Yet, selective logging significantly increased SOC (*F*_2,6_ = 6.100, *p* < 0.05, Figure 3A), SAN (*F*_2,6_ = 101.647, *p* < 0.01, Figure 3C), SAP (*F*_2,6_ = 9.815, *p* < 0.05, Figure 3E), SAK (*F*_2,6_ = 21.151, *p* < 0.01, Figure 3G), and SBD (*F*_2,6_ = 18.609, *p* < 0.01, Figure 3J). Selective logging, especially as part of *LCT*, increased SAN and SBD.

**Figure 4:**
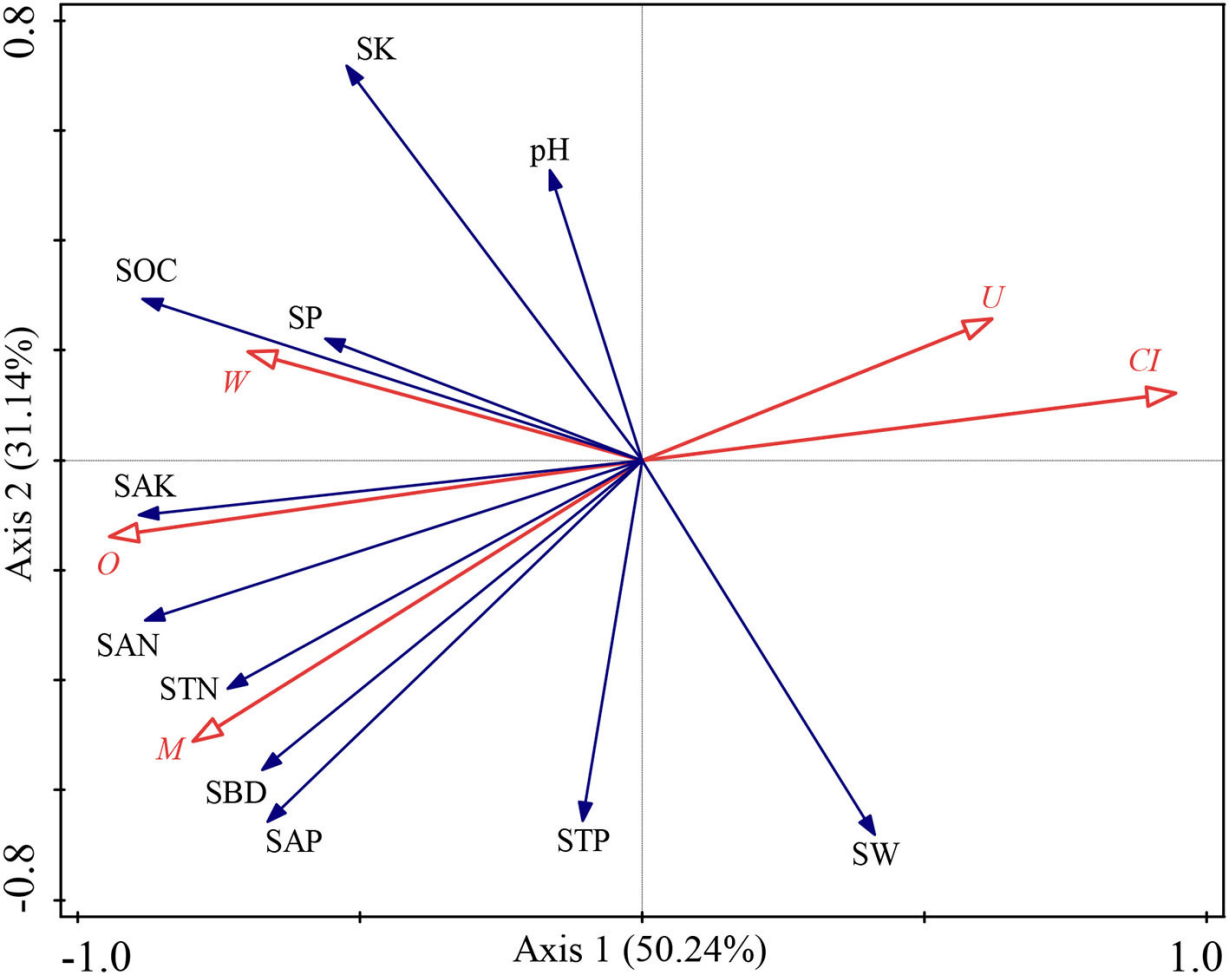
Redundancy analysis (RDA) among the stand spatial structure characteristic indexes and environmental factors. The angle between the two indicators represents a significant and positive relationship (acute angle, < 90°) or negative relationship (obtuse angle, >90°). The length of the arrows is proportional to the magnitude of the standardized path coefficients (i.e., the strength of the relationship). The stand spatial structure parameters are *M*, mingling index; *W*, uniform angle index; *CI*, competition index; *U*, neighborhood comparison; and *O*, opening degree. The soil parameters are SOC, soil organic carbon; STN, soil total nitrogen; SAN, soil alkaline nitrogen; STP, soil total phosphorus; SAP, soil available phosphorus; SK, soil total potassium; SAK, soil available potassium; SW, soil water content; pH, soil pH; SBD, soil bulk density; SP, soil porosity.

The SW was significantly and negatively correlated with *W* (*p* < 0.05, Supplementary Figure 1). The SBD was significantly and positively correlated with *M* and *O* (*p* < 0.05, Supplementary Figure 1), yet vice versa for *CI* (*p* < 0.05, Supplementary Figure 1). The SOC was significantly and positively correlated with *W* and *O* (*p* < 0.05, Supplementary Figure 1), but negatively correlated with *CI* (*p* < 0.05, Supplementary Figure 1). The STN was significantly and positively correlated with *M* (*p* < 0.05, Supplementary Figure 1). The SAN was significantly and positively correlated with *M, W*, and *O* (*p* < 0.05, Supplementary Figure 1), but negatively correlated with *CI* and *U* (*p* < 0.05, Supplementary Figure 1). The SAP was significantly and positively correlated with *M* (*p* < 0.05, Supplementary Figure 1). The SAK was significantly and positively correlated with *M* and *O* (*p* < 0.05, Supplementary Figure 1), but negatively correlated with *CI* and *U* (*p* < 0.05, Supplementary Figure 1). Furthermore, we also explored the effects of the stand spatial structure characteristic indexes on environmental factors through an RDA (Figure 4). The interpretation of RDA's first and second axes amounted to 50.24 and 31.14%, respectively (Figure 4), for a combined contribution totaling 81.38%. Specifically, the *CI* was the main factor driving variation in the environmental factors (*F* = 5.9, *p* = 0.002 < 0.01, Figure 4), for which the explanatory and contributing percentages were 45.9 and 51.1%, respectively. The competitive relationship of the stand forest accounted for most of the environmental factors. Second, the corresponding percentages for the *M* in the first and second axes were 18.9 and 21.1%, respectively (*F* = 3.2, *p* = 0.06 > 0.05), which indicated that the diversity of tree species also affected soil environmental factors although the effect was insignificant. Accordingly, the joint contribution by the *CI* and *M* surpassed that of all other stand spatial structure characteristic indexes combined, differentiating these two as the major factors affecting the soil physicochemical parameters.

### The growth, biomass accumulation, and biomass allocation to seedlings

**Figure 5:**
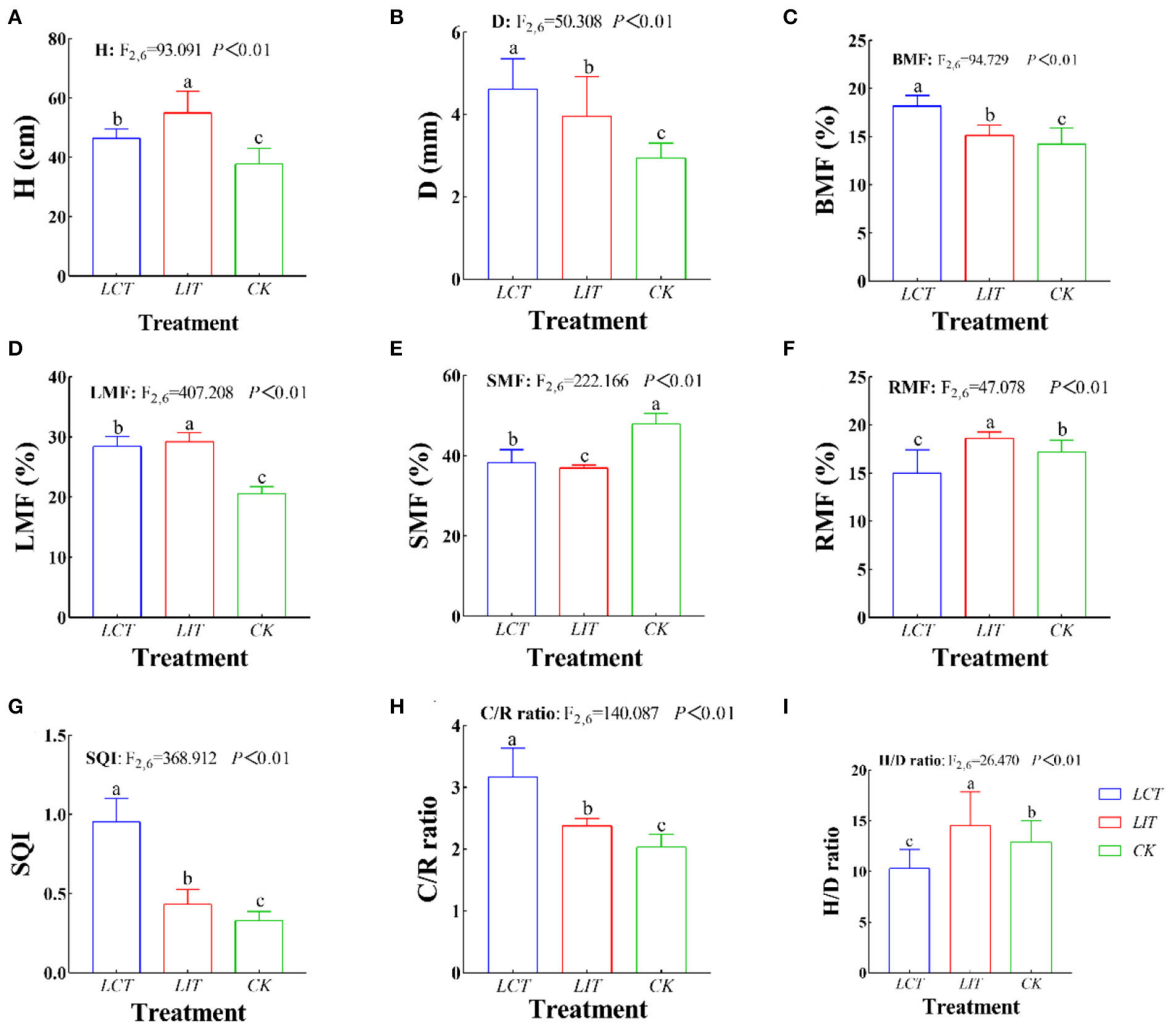
The growth and biomass allocation of *Pinus massoniana Lamb*. The logging methods are *LCT*, logging of the competitive trees; *LIT*, logging of the inferior trees; *CK*, unlogged control. The growth indexes are H, plant height (cm); D, ground-diameter (mm); BMF, branch mass fraction (%); LMF, foliage mass fraction (%); SMF, stem mass fraction (%); RMF, root mass fraction (%); C/R ratio, crown-root ratio; H/D ratio, height-diameter ratio; SQI, seedling quality index. Data shown are the mean ± standard deviation (*n* = 3). Different lowercase letters indicate significant differences among the treatments (*p* < 0.05) in **(A–I)**.

The plant height (H), ground-diameter (D), and biomass of the natural regeneration seedlings of *P. massoniana* under different treatments are shown in Supplementary Tables 4, 5 and Figure 5. The H, D, and biomass among the three treatments were 46.38 cm, 3.84 mm, and 8.21 g, respectively (Figure 5A). The biomass among organs was roughly as follows: stem > foliage > branch ≈ root (Figure 5B).

After selective logging, H (*F*_2,6_ = 93.091, *p* < 0.01, Figure 5A), D (*F*_2,6_ = 50.308, *p* < 0.01, Figure 5B), branches biomass fraction (BMF) (*F*_2,6_ = 94.729, *p* < 0.01, Figure 5C), foliage biomass fraction (LMF) (*F*_2,6_ = 407.208, *p* < 0.01, Figure 5D), stems biomass fraction (SMF) (*F*_2,6_ = 222.166, *p* < 0.01, Figure 5E), roots biomass fraction (RMF) (*F*_2,6_ = 47.078, *p* < 0.01, Figure 5F), SQI (*F*_2,6_ = 368.054, *p* < 0.01, Figure 5G), C/R ratio (*F*_2,6_ = 139.489, *p* < 0.01, Figure 5H), and H/D ratio (*F*_2,6_ = 26.475, *p* < 0.01, Figure 5I) of 3-year-old seedlings were significantly different from the seedling in the unlogged treatment (*p* < 0.05). In *LCT*, the D and biomass of organs were the largest (*p* < 0.05), while *LIT* significantly improved the H (*p* < 0.05).

### The relationships among biomass allocation, spatial structure characteristic indexes, and environment factors

**Figure 6:**
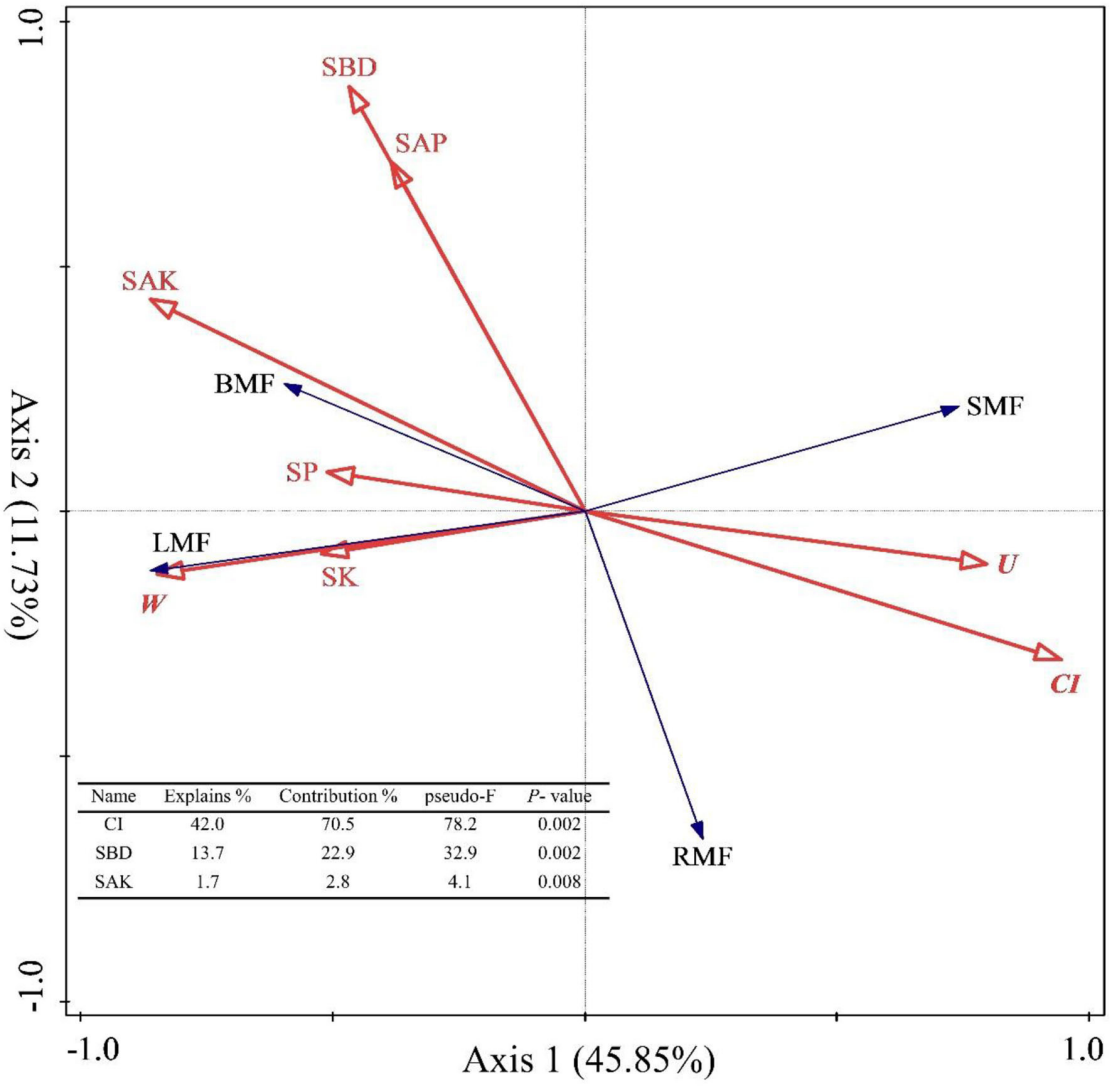
Redundancy analysis (RDA) among the stand spatial structure characteristic indexes, environmental factors, and biomass allocation of organs. The angle between the two indicators represents a significant and positive relationship (acute angle, < 90°) or negative relationship (obtuse angle, >90°). The length of the arrows is proportional to the magnitude of the standardized path coefficients (i.e., the strength of the relationship). The growth indexes are C/R ratio, crown-root ratio; H/D ratio, height-diameter ratio; and SQI, seedling quality index. The stand spatial structure parameters are *M*, mingling index; *W*, uniform angle index; *CI*, competition index; *U*, neighborhood comparison; and *O*, opening degree. The soil parameters are SOC, soil organic carbon; STN, soil total nitrogen; SAN, soil alkaline nitrogen; STP, soil total phosphorus; SAP, soil available phosphorus; SK, soil total potassium; SAK, soil available potassium; SW, soil water content; pH, soil pH; SBD, soil bulk density; SP, soil porosity.

The spatial structure characteristic indexes and environmental factors affected the biomass allocation to organs. First, we used Pearson correlation to analyze how these factors are related to biomass allocation to organs. The results showed that the BMF was significantly and positively correlated with SOC, STN, SAN, STP, SAP, SAK, SBD, SP, *M, W*, and *O* (*p* < 0.05, Supplementary Figure 2), but significantly and negatively correlated with SW, *CI*, and *U* (*p* < 0.05, Supplementary Figure 2). The LMF was significantly and positively correlated with SOC, STN, SAN, SAP, SK, SBD, SP, *M, W*, and *O* (*p* < 0.05, Supplementary Figure 2), but negatively correlated with SW, *CI*, and *U* (*p* < 0.05, Supplementary Figure 2). The SMF was significantly and positively correlated with SW, *CI*, and *U* (*p* < 0.05, Supplementary Figure 2), but significantly and negatively correlated with SOC, STN, SAN, SK, SAK, SP, *M, W*, and *O* (*p* < 0.05, Supplementary Figure 2). The RMF was significantly and positively correlated with *CI* and *U* (*p* < 0.05, Supplementary Figure 2), but significantly and negatively correlated with SOC, STN, SAN, STP, SAP, SAK, SW, SBD, *M*, and *O* (*p* < 0.05, Supplementary Figure 2). In other words, the biomass allocation to organs depended on the stand spatial structure and environmental factors, and strategies were adopted for heterogeneous adaptation and growth, thereby resulting in the separation of allocation patterns among the organs. Then, we also explored the effects of the stand spatial structure characteristic indexes and environmental factors on the biomass allocation to organs through an RDA (Figure 6). The interpretation of RDA's first and second axes were 45.85 and 11.73%, respectively (Figure 6) for a combined contribution of 57.59%. Specifically, the *CI* was the main factor driving variation in the biomass allocation (*F* = 78.2.5, *p* = 0.002 < 0.01, Figure 6), in which the explanatory and contribution percentages were 42.0 and 70.5%, respectively. The competitive relationship of the stand forest was the basis for biomass allocation. Second, the corresponding percentages for the SBD in the first and second axes were 13.7 and 22.9%, respectively (*F* = 32.9, *p* = 0.002 < 0.01, Figure 6), which indicated that the number of soil pores and soil solids were smooth and entered the soil to obtain the necessary water and nutrient resources during the two key periods of the settlement and growth of seedlings. At the same time, the explanatory and contribution percentage of the SAN was 1.7 and 2.8%, respectively (*F* = 4.1, *p* = 0.008 < 0.01, Figure 6), which indicated that the supply of the available nitrogen at the seedling stage had a strong effect on the growth and biomass allocation to seedlings. The joint contribution by these three factors was far higher than other parameters combined, distinguishing the three as the main and significant factors affecting the growth and biomass allocation (*p* < 0.05).

**Figure 7:**
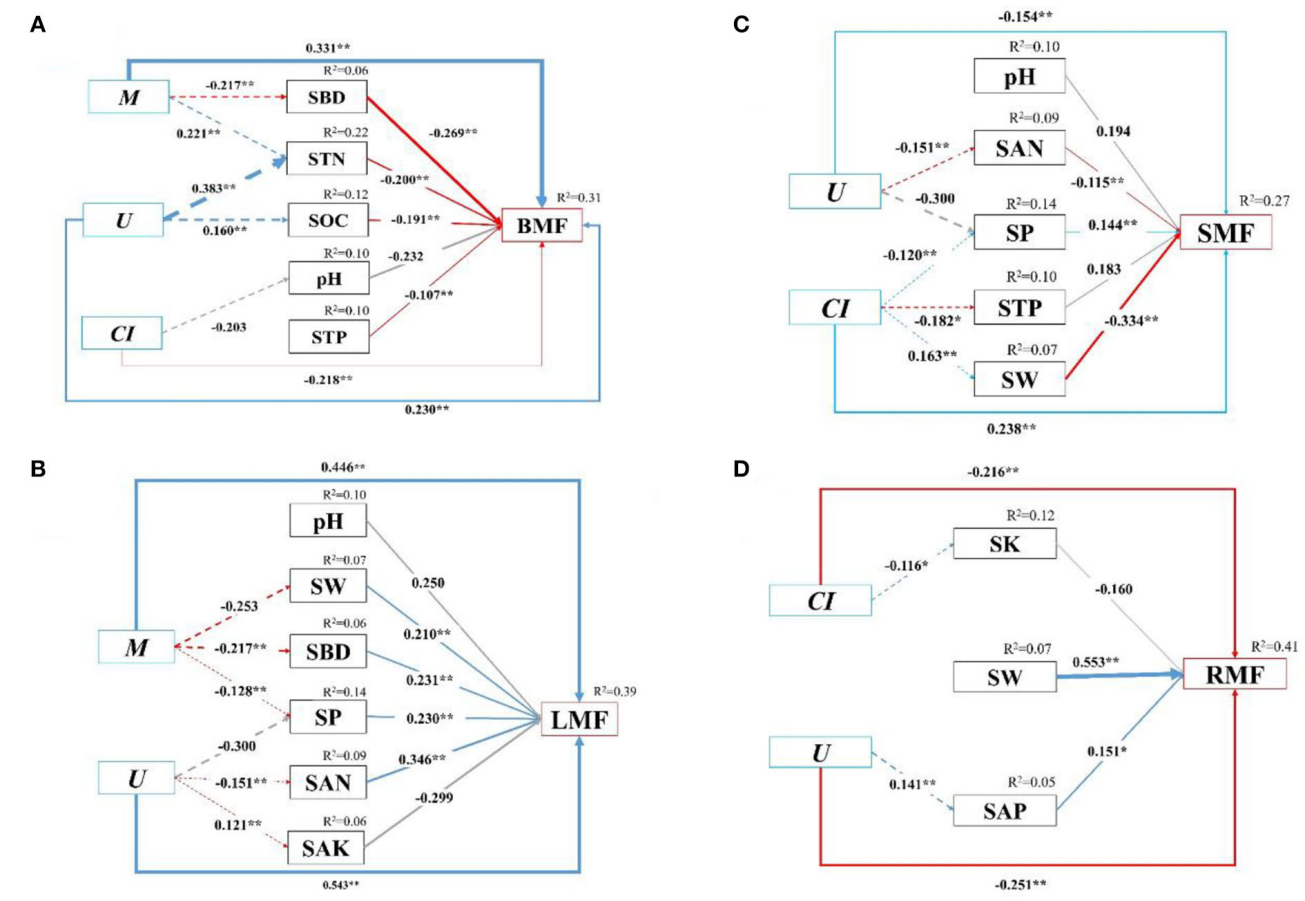
**(A–D)** The structural equation model (SEM) among biomass allocation of organs, stand spatial structure characteristic indexes, and environmental factors. Chi-square (χ^2^) = 97.72, DF = 112, Chi/DF = 0.873, GFI = 0.948, NFI = 0.938, PGFI = 0.672, RMSEA = 0.015, AIC = 363.720, *p* = 0.102. The solid and dashed arrow are the direct and indirect pathways, respectively. The blue and red arrow represent significant positive and negative effects, respectively. The gray dashed arrows represent no significant pathways. The thickness of the arrows is proportional to the magnitude of the standardized path coefficients. The arrow width is proportional to the strength of the relationship. The growth indexes are C/R ratio, crown-root ratio; H/D ratio, height-diameter ratio; and SQI, seedling quality index. The stand spatial structure parameters are *M*, mingling index; *W*, uniform angle index; *CI*, competition index; *U*, neighborhood comparison; and *O*, opening degree. The soil parameters are SOC, soil organic carbon; STN, soil total nitrogen; SAN, soil alkaline nitrogen; STP, soil total phosphorus; SAP, soil available phosphorus; SK, soil total potassium; SAK, soil available potassium; SW, soil water content; pH, soil pH; SBD, soil bulk density; SP, soil porosity.

Furthermore, we quantified the relative contribution percentages of the stand spatial structure characteristic indexes and environmental factors to the biomass allocation to different organs with the SEM (*p* = 0.102 > 0.05, χ^2^ = 97.72, DF = 112, Chi/DF = 0.873, GFI = 0.948, NFI = 0.938, PGFI = 0.672, RMSEA = 0.015, AIC = 363.720, Figure 7). The models could explain 15.2–78.6% of the variance in the biomass allocation to organs. For all three treatments, the stand spatial structure characteristic indexes and environmental factors had direct effects on the biomass allocation to organs, while the stand spatial structure characteristic indexes had indirect effects on the biomass allocation through environmental factors. Specifically, the *M* had significant and indirect effects on the BMF through SBD (−0.269, *p* < 0.01) and STN (−0.200, *p* < 0.01), and the *U* had a significant and indirect effect on the BMF through STN (−0.200, *p* < 0.01) and SOC (−0.191, *p* < 0.01). The *M* had negative directive effects on SBD (−0.217, *p* < 0.01) instead of STN (0.221, *p* < 0.01), whereas the *U* had positive directive effects on STN (0.383, *p* < 0.01) and SOC (0.160, *p* < 0.01; Figure 7A). The *M* had significant and indirect effects on the LMF through SW (0.210, *p* < 0.01), SBD (0.231, *p* < 0.01), and SP (0.230, *p* < 0.01), and the *U* had significant and indirect effects on the LMF through SP (0.231, *p* < 0.01), SAN (0.346, *p* < 0.01), and SAK (−0.299, *p* > 0.05). The *M* had negative directive effects on SW (−0.253, *p* < 0.01), SBD (−0.217, *p* < 0.01), and SP (−0.128, *p* < 0.01). Simultaneously, the *U* had a positive directive effect on SAK (0.121, *p* < 0.01), a negative directive effect on SAN (−0.151, *p* < 0.01), and an insignificant effect on SP (−0.300, *p* > 0.05; Figure 7B). The *U* had significant and indirect effects on the SMF through SAN (−0.115, *p* < 0.01) and SP (0.144, *p* < 0.01), and the *CI* had significant and indirect effects on the SMF through SP (0.144, *p* < 0.01), STP (0.183, *p* < 0.01), and SW (−0.334, *p* < 0.01). The *U* had a negative directive effect on SAN (−0.1515, *p* < 0.01), but had no significant effect on SP (−0.300, *p* > 0.05). The *CI* had negative directive effects on SP (−0.120, *p* < 0.01) and STP (−0.182, *p* < 0.05), whereas it had a positive directive effect on SW (0.163, *p* < 0.01; Figure 7C). The *U* had a significant and indirect effect on the RMF through SAP (0.151, *p* < 0.01). The *U* had a positive effect on SAP (0.141, *p* < 0.01; Figure 7D).

Overall, the competition within the forest affected the growth and biomass allocation in the naturally regenerating seedlings by adjusting environmental factors.

## Discussion

### The influence of logging on spatial structure characteristic indexes

Compared to the non-spatial structure, the spatial structure of the stand contains the positional information of trees and the spatial (horizontal and vertical direction) relationships among neighboring trees. This information directly or indirectly determines the competition intensity among neighboring trees and the effects of the growth-related environmental factors, which have a great impact on the growth and development of remaining trees, as well as the growth and biomass allocation during natural regeneration (Liu N. et al., 2021; Dong et al., 2022). The mingling index (*M*) and uniform angle index (*W*) are the main horizontal spatial structure indexes that help maintain the diversity benefits and ecological functions (Dong et al., 2022). In this study, the *M* increased upon *LCT*, but not in *LIT*. This result is closely related to the logging method (Boivin-Dompierre et al., 2017; Piiroinen et al., 2017; Yin et al., 2022). Specifically, *P. massoniana* is cut in *LCT*, while shorter species of other tree species (such as *C. funebris, T. sinensis*, and *B. papyrifera*) are preserved as the ecological crop-management trees, which were cut due to being classified as inferior trees (V-trees, IV-trees, and parts of III-tree) in *LIT*. Furthermore, in *LCT*, the forest stand may promote the circulation rate of nitrogen, phosphorus, and potassium in soil due to the increased diversity of tree species and the complex relationships among neighboring trees (Dánescu et al., 2018), thereby affecting the growth and biomass allocation during natural regeneration. After logging, the horizontal distribution pattern of forest trees was transformed from a very regular or regular distribution to a random distribution, which has previously been confirmed by many studies (Hui et al., 2018; Dong et al., 2022). Weakening or removing the competition from big trees expands the space range of growth and increases the availability of light, temperature, and water in patchy areas, which is beneficial to the growth of seedlings and biomass allocation in them.

In addition, the openness degree (*O*) was used to represent the vertical spatial structure index of stands (Gadow et al., 2012). We found that the *O* of forest stands increased for *LCT* or *LIT*. The intensity of light was stronger in the forest understory as compared to the unlogged control, which was also consistent with the conclusions of previous studies (Gadow et al., 2012; Lai et al., 2019; Yin et al., 2022). The sub-dominant trees that competed with the dominant trees were cut in *LCT*; these competitive trees were present in the upper layers of the stand, thus the *O* of *LCT* must be greater than that of the *LIT*, in which only the lower-layer inferior trees and a small amount of the upper layers of trees were cut. Therefore, the light requirements of *P. massoniana* at the seedling stage could potentially be better met in *LCT*. Simultaneously, the competition index (*CI*) and neighborhood comparison (*U*) were used to determine the competitive situation of the neighboring trees (Hui et al., 2018; Liu N. et al., 2021; Dong et al., 2022). Light, nutrients, and spatial extent are among the most important environmental factors for the growth of trees at the stage of the near-mature forest (Lai et al., 2019). In summary, logging reduced the intensity of the competition by light (the space of canopy), soil nutrients and water, and the size and dominance degree among neighboring trees, which was more helpful to naturally regenerating seedlings and the growth of young trees, especially in LCT.

### The influence of logging on soil physiochemical properties

The stand spatial structure controls the structure and function of forest ecosystems (Dong et al., 2022). The forest gaps formed after logging change the environmental factors (such as temperature, water, and light), as well as the nutrient release and cycling due to forest litter, which affected soil physiochemical properties in the forest (Yin et al., 2022). Simultaneously, the structure determined the function, thus there must be differences among heterogeneous logging methods. In this study, the soil bulk density (SBD) increased significantly in *LCT*, but not in *LIT*. This observation was different from the conclusions of Tchiofo Lontsi et al. (2019) and Zhou et al. (2021) that the SBD does not change after logging. There might be two reasons: first, their harvesting operations comprised heavy traffic that increased soil compaction (Tchiofo Lontsi et al., 2019), leading to a decrease or no-significant change in SBD. Second, logging is beneficial to improving the activities of macrofauna (earthworms and nematodes) and soil enzymes related to the transformation of nitrogen and phosphorus in the upper layer of the forest, which could also help in soil loosening (Qiu et al., 2020; Yin et al., 2022). The work by Tu et al. (2022) emphasized that these biological phenomena were related to factors, such as light (openness degree, *O*) and the diversity of species (mingling index, *M*), therefore, SBD increased significantly in *LCT*. In addition, we found that the soil water content (SW) decreased significantly in *LIT* but did not change in *LCT*. Similar results have been presented in some other research reports (Zhang et al., 2018; Lull et al., 2020; Zhou et al., 2021). Other studies have contrarily noted that logging increased SW (Cheng et al., 2015; Qiu et al., 2020; Fernández et al., 2021). These differences might result from two aspects: first, the effect of the thinning in SW depends on the density of the canopy and the intensity of the rainfall, where mild rains are retained by the canopy and do not reach the soil, while heavy rains reach the soil even through dense canopies, where higher soil temperature increases SW evaporation (Zhang et al., 2018). Second, decreased SW may be related to the intensity of the thinning, which might be attributed to the wind. The intensity of the thinning increased the movement of the wind and accelerated the soil evaporation and decreased SW (Lull et al., 2020; Zhou et al., 2021).

Logging not only changed soil physical properties, but also affected soil chemical properties (Cheng et al., 2015; Qiu et al., 2020; Yin et al., 2022). Our results showed that forest logging did not have significant effects on the soil pH (pH), which was the same as many studies (Qiu et al., 2020; Fernández et al., 2021). On the one hand, the foraging preference of *P. massoniana* is ${\text{NH}}_{4}^{+}$, which causes H^+^ to be replaced by the base ions on the soil cation exchange site in soil solution, thereby increasing the concentration of H^+^. On the other hand, the H_2_CO_3_ formed by the reaction of CO_2_ from the decomposition of the soil matrix and forest litter in the presence of water may weaken or even counteract this acidification process through the buffer reaction. Additionally, logging has no significant effects on the soil total phosphorus (STP). However, a meta-analysis performed by Zhou et al. (2021) suggested that logging has a significant effect on STP, which might be lined with climatic factors and forest types (Bai et al., 2017) and could be affected by the intensity of the thinning (Zhou et al., 2019). Logging could also increase soil available nitrogen (SAN) and available phosphorus (SAP), which is in line with the conclusions of Cheng et al. (2015) and Qiu et al. (2020). This might be because logging changed the microclimate in the forest, thereby promoting the releasing and cycling of nutrients (García-Palacios et al., 2013). Logging has a significant and positive effect on the soil organ carbon (SOC) and total nutrient (STN). The meta-analysis from Zhou et al. (2021) also uncovered that logging may strengthen SOC and STN, while other reports have underlined that the SOC and STN can reduce (Tchiofo Lontsi et al., 2019) or even remain unchanged (Bai et al., 2017) after logging. These results were mainly related to a reduction of the forest litter since the major source of SOC and STN is the forest litter (Lull et al., 2020). In this study, regardless of *LCT* or *LIT*, the fallen trees were kept in the native place in the form of forest litter, which increased the available amount of carbon and nitrogen for decomposition and return to the soil in the short-term (Lull et al., 2020; Qiu et al., 2020). Simultaneously, changes in light, water, and heat conditions attributed to logging also promoted the decomposition rates of the forest litter (Yin et al., 2022).

Furthermore, we found that *CI* was the main effect on soil physiochemical properties. After logging, the opened canopy of the stand with more light and higher temperature increases the decomposition rate of forest litter and improves the release rate of nutrients (Lull et al., 2020; Qiu et al., 2020; Zhou et al., 2021). Simultaneously, the fallen trees that remain on the stand serve as forest litter, increasing the amount of carbon and nitrogen to decompose and return to the soil in a short-term of 2 years (Lull et al., 2020; Yin et al., 2022). The *M* also has a positive, albeit insignificant, effect on soil physiochemical properties. This might result from there being fewer companion trees in this study, especially, the species and number of broad-leaved trees, thereby producing a lower amount of mixed forest litter, which did not have a significant promotional effect to quicken nutrient release and cycling (Tie et al., 2022; Wang et al., 2022a).

### The influence of logging on the regeneration and growth of seedlings under logging

As compared to the unlogged sites, in both *LCT* or *LIT*, the number, height, and ground-diameter of the regeneration significantly increased. This is in line with some other research reports. For example, Rivett et al. (2016) found that logging had either a neutral or positive impact on the density of seedlings of timber species as compared to the unlogged forest, indicating that it was probably a response to a moderate increase in light levels. de Carvalho et al. (2017) also found that the number of regenerating seedlings gradually increased over time with larger canopy openness and lower soil bulk density. The work by Zhao et al. (2021) also emphasized that selective logging is beneficial to regeneration and has a positive effect on the plant height and ground-diameter of regenerating seedlings. Yet, these two methods (*LCT* and *LIT*) showed a significant difference in the extent of survival and growth of the naturally regenerated seedlings. There might be three reasons: first, based on the stochastic body-stability hypothesis (Hui et al., 2018), the *W* that can construct many stable random bodies with a lower degree of squeezing and less survival pressure of seedlings, changed more in the *LCT* than in the *LIT*; therefore, the chances to grow stably were higher in *LIT*. Second, the *O* of the canopy was heterogeneous (de Carvalho et al., 2017; Liang and Wei, 2021). Specifically, the *LCT* could enable the crop-management tree (or the central tree) to reach sunlight from two or more directions in the canopy. Nonetheless, as shorter trees were cut in *LIT*, incoming sunlight did not change. More light is an important factor in nutrient cycling in the forest and is more helpful to the growth and development of seedlings. Third, the competition was unequal after logging in the forest (Piiroinen et al., 2017). In *LCT*, the competition could be smaller in the forest, which could reduce or even eliminate the growth suppression effect of the big trees on seedlings. In general, the lower the intensity of logging, the more conducive to the natural regeneration and growth of seedlings with logging of the competitive trees.

In this study, biomass accumulation and allocation during natural regeneration were also affected by the treatments. There might be four reasons: first, the differences among treatments might be related to the turnover rate of the organs (McCarthy and Enquist, 2007; Sevillano et al., 2016; Umaña et al., 2021). The turnover rate of the foliage and roots was generally the highest, while that of the stems was the slowest. Second, these results may also be interrelated to function equilibrium (Cella Pizarro and Bisigato, 2010; Santiago et al., 2012; Yan et al., 2016; Bebre et al., 2021; Liu R. et al., 2021). Vegetative growth is the most important process at the seedling stage when resource limitation feedback among organ growth ensures an increase in the uptake of the most limiting factor to achieve some “balanced growth.” This reflects the organ's trade-off strategy for the competition. Stems are the most in demand for resources, followed by foliage and roots, while the demand is lowest for branches. Third, it may be caused by the increase in nutrient absorption and SBD. For instance, Saldaña-Acosta et al. (2009), Sevillano et al. (2016), Bebre et al. (2021), and Liu R. et al. (2021) found that plants may adopt a series of growth strategies by increasing the height and improving the branch and stem biomass to indirectly expand the range of leaf area to obtain more light that provides protection, as well as opportunities for the growth and development of seedlings by adapting to low light conditions. Fay et al. (2015) and Santiago et al. (2012) also confirmed that the addition of nitrogen, phosphorus, and potassium fertilizers helps adjust the distribution patterns of biomass among organs. These nutrients would preferably be allocated to the stems of the above-ground parts to support the foliage. Sahoo et al. (2021) also found that the foliage biomass and above-ground parts would also become heightened with an increase in the soil nitrogen content. Fourth, these results might also be caused by the forest stand spatial structure changes. The targets and strategies of forest management were different in these two treatments, whereby *LCT* emphasized the *M, W*, and *O* for which the growth of seedlings was required in the forest (Li et al., 2020). This manifested as an increase in the ground-diameter. However, in *LIT*, the number of plants in the forest was reduced (Boivin-Dompierre et al., 2017; Piiroinen et al., 2017; Bose et al., 2018), which had a more direct effect on the release of competitive pressure than the light intensity. Therefore, the seedlings needed to adapt to the survival strategy of obtaining light, i.e., the more the stem biomass, the greater the plant height. In the unlogged treatment, the growth of the seedling was not only suppressed by the stand spatial structure but also the competitive pressure in the environment, which resulted in poor ground-diameter and height of the regenerated seedlings.

### The influence of spatial structure characteristic indexes and environmental factors on seedling biomass allocation

To understand the determinants of stand spatial structure and environment components on biomass allocation to the different organs, we tested the direct or indirect effects of the stand structure characteristic indexes and environmental factors on the growth and biomass allocation. In this study, the *CI* in the forest stand was the main and direct factor affecting the regeneration, growth, and biomass allocation in seedlings, which was similar to the results of Poorter et al. (2012), Zhou et al. (2018), and Rehling et al. (2021). In addition, our results indicated that spatial structure characteristic indexes have an indirect effect on the biomass allocation to organs through environmental factors. Selective logging reduces the number of big trees and increases the relative distance of the remaining trees (Hui et al., 2018; Wan et al., 2019), thereby there is reduced pressure by competition, leaving a safe habitat for the settlement and growth of seedlings. These safe conditions offer increased light as well as release and recycling of nutrients in the forest litter, which are key factors for the successful germination, growth, and development of seedlings. To a certain extent, forming spatial gaps in the forest could improve and supplement the light intensity required by the growth of seedlings (Li et al., 2020). However, compared with the *LCT*, stronger *LIT* might open up the forest canopy, allowing more light in the forest (Boivin-Dompierre et al., 2017; Piiroinen et al., 2017; Bose et al., 2018). At the same time, we found that there were more seedlings where the interfering trees were removed in the *LCT*. Furthermore, the SBD was also an important and directly related environmental factor, which was similar to the results of Sun et al. (2019). During the two key periods of plant settlement and growth, the composition of soil and the size of soil pores were the main constraints on the roots of a seedling to enter smoothly into the soil medium. After selective logging, the available nutrients (such as nitrogen, phosphorus, and potassium) improved, but that had less effect on the growth and biomass allocation in the seedling. This was different from the results of Wright et al. (2011), Santiago et al. (2012), and Fay et al. (2015). We speculate that the demand for these nutrients was not enough during the growth stage of the seedlings, but these influencing factors and their effects might change over time.

Furthermore, we also found that the factors driving biomass allocation to the different organs were divergent, which was similar to the results of Umaña et al. (2021). The resource utilization and functional zoning in different organs were heterogeneous; therefore, the biomass allocation could operate independently and adopt different adaptation and growth strategies, thereby resulting in the separation of the biomass allocation among the organs. Contemporarily, we were surprised to find out that the foliage, as the most important photosynthetic tissue organ, was more affected by the spatial structure, such as competition index, instead of soil nutrients such as STN, STP, and SK. It might be that these nutrients were not so limited in this forest to restrict the growth of plants (de Carvalho et al., 2017). Specifically, *P. massoniana*, as an ectomycorrhizal tree species (Li et al., 2020), can increase nutrient absorption through the mycorrhiza and expand the absorption area of its root system to meet its own growth needs.

## Conclusion

Whether the competitive trees or the inferior trees were logged, the number, height, and ground-diameter of regenerated seedlings had significantly increased (*p* < 0.05). Upon logging the competitive trees, seedlings adopted a survival strategy that improved their branch biomass and increased the ground-diameter, crown-root ratio, and seedling quality index. Seedlings took a series of survival strategies that allocated more biomass to foliage and roots and increased plant height and height-diameter ratio upon logging of the inferior trees. In the unlogged forests, stem biomass increased in seedlings to absorb water and nutrients and more light was consumed in photosynthesis. Biomass allocation to different organs was independent and took the strategies of heterogeneity adaptation and growth, thereby resulting in the separation of the allocation patterns among organs. Selective logging changed the spatial structure of the forest stand, which had a significant and direct impact on environmental factors, while promoting the growth of the natural regeneration and biomass allocation to organs. The competition between the big trees in the forest and the naturally regenerating seedlings was the main and direct factor affecting biomass allocation (*p* < 0.05); it had an indirect effect on the biomass allocation to organs through environmental factors. Meanwhile, soil nutrient factors had less effect on the regeneration, growth, and biomass allocation to seedlings (*p* > 0.05). In summary, logging of the competitive trees has significant and indirect effects on SOC, SAN, and SBD by reducing the *CI* of the stand, affecting the regeneration and growth of seedlings. These findings are of great significance to the sustainable development of forest stands of *P. massoniana*.

## Data availability statement

The original contributions presented in the study are included in the article/Supplementary material, further inquiries can be directed to the corresponding author.

## Author contributions

WH: methodology, data curation, formal analysis, writing—original draft, and writing—review and editing. YW, XWa, XWe, TL, and MY: data curation and writing—original draft. GC, KZ, GH, XL, and CF: writing—review and editing. All authors contributed to the study conception and design. All authors contributed to the article and approved the submitted version.

## Funding

This study was supported by the German Government loans for Sichuan Forestry Sustainable Management (Grant No. G1403083) and the Key Sci-Tech Project of the 13th 5-year Plan of China (Grant No. 2017YFD060030205).

## Conflict of interest

The authors declare that the research was conducted in the absence of any commercial or financial relationships that could be construed as a potential conflict of interest.

## Publisher's note

All claims expressed in this article are solely those of the authors and do not necessarily represent those of their affiliated organizations, or those of the publisher, the editors and the reviewers. Any product that may be evaluated in this article, or claim that may be made by its manufacturer, is not guaranteed or endorsed by the publisher.
