# Supplementary material for: Stand structure adjustment influences the biomass allocation in naturally generated Pinus massoniana seedlings through environmental factors
Source: Front Plant Sci. 2022 Oct 28;13:997795. doi: 10.3389/fpls.2022.997795 (PMC9650532; doi:10.3389/fpls.2022.997795)
Supplement: Supplementary file 1 [file Data_Sheet_1.zip › Supplementary Images.DOCX]

Supplementary Figures


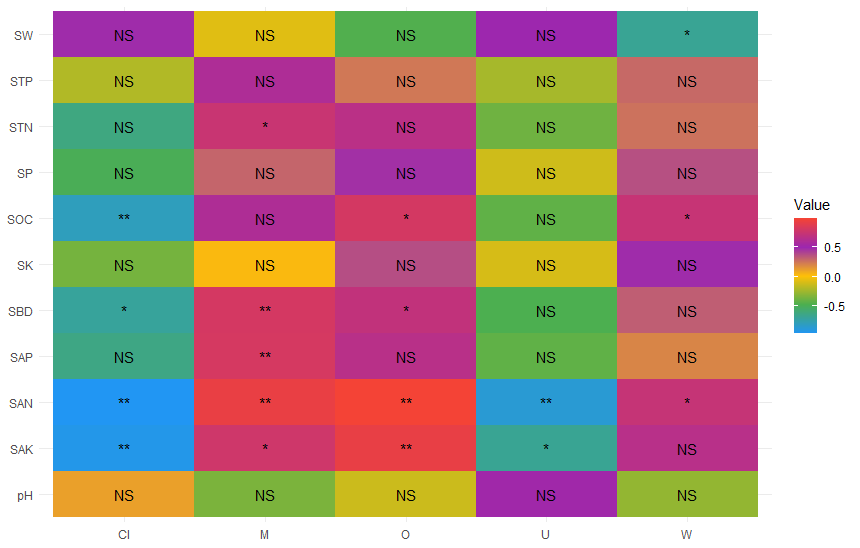


**Supplementary Figure 1. Correlation coefficients between the stand spatial structure characteristic indexes and environmental factors.**

**Note:** The red and blue colors denote significant positive and negative effects, respectively. The color intensity is proportional to the magnitude of the Pearson correlation. The darker the color, the greater the strength of the relationship, and vice versa. The stand spatial structure parameters are ***M***, mingling index; ***W***, uniform angle index; ***CI***, competition index; ***U***, neighborhood comparison; ***O***, opening degree. The soil parameters are **SOC**, soil organic carbon; **STN**, soil total nitrogen; **SAN**, soil alkaline nitrogen; **STP**, soil total phosphorus; **SAP**, soil available phosphorus; **SK**, soil total potassium; **SAK**, soil available potassium; **SW**, soil water content; **pH**, soil pH; **SBD**, soil bulk density; **SP**, soil porosity. The NS means not significant. The * and ** indicate significant at *p* < 0.05 and *p* < 0.01, respectively.


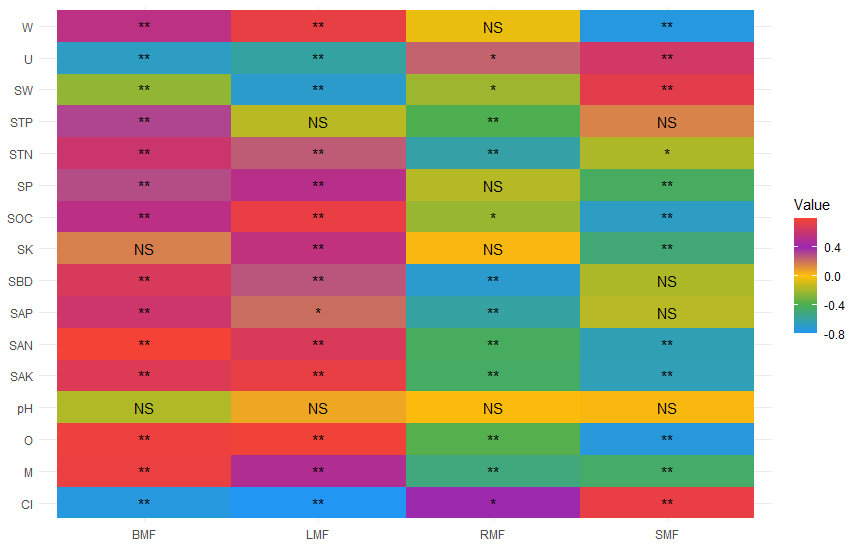


**Supplementary Figure 2. Correlation coefficients between the stand spatial structure characteristic indexes and environmental factors and the biomass allocation of organs.**

**Note:** Correlation coefficients between stand spatial structure characteristic indexes and environmental factors. The red and blue colors denote significant positive and negative effects, respectively. The color intensity is proportional to the magnitude of the Pearson correlation. The darker the color, the greater the strength of the relationship, and vice versa. The growth indexes are **C/R ratio**, crown-root ratio; **H/D ratio**, height-diameter ratio; **SQI**, seedling quality index. The stand spatial structure parameters are ***M***, mingling index; ***W***, uniform angle index; ***CI***, competition index; ***U***, neighborhood comparison; ***O***, opening degree. The soil parameters are **SOC**, soil organic carbon; **STN**, soil total nitrogen; **SAN**, soil alkaline nitrogen; **STP**, soil total phosphorus; **SAP**, soil available phosphorus; **SK**, soil total potassium; **SAK**, soil available potassium; **SW**, soil water content; **pH**, soil pH; **SBD**, soil bulk density; **SP**, soil porosity. The NS means not significant. The * and ** indicate significant differences at *p* < 0.05 and *p* < 0.01, respectively.
