# Supplementary material for: Stand structure adjustment influences the biomass allocation in naturally generated Pinus massoniana seedlings through environmental factors
Source: Front Plant Sci. 2022 Oct 28;13:997795. doi: 10.3389/fpls.2022.997795 (PMC9650532; doi:10.3389/fpls.2022.997795)
Supplement: Supplementary file 1 [file Data_Sheet_1.zip › Supplementary Tables.DOCX]

**Supplementary Table 1**

The number and growth of the three-year-old seedlings of *Pinus massoniana* Lamb*.*

| Treatment | Number (plant·hm^-1^) | Height (cm) | Ground-diameter (mm) |
| --- | --- | --- | --- |
| *LCT* | 300±17a | 44.13±5.41b | 4.63±0.59a |
| *LIT* | 289±10a | 51.24±4.72a | 3.92±0.42b |
| *CK* | 239±10b | 41.42±2.91c | 3.08±0.56c |
| *Al^la^* | 276±30 | 44.73±5.85 | 3.64±0.75 |

**Note:** The logging methods are ***LCT***, logging of the competition trees; ***LIT***, logging of the inferior trees; ***CK***, unselected-logging; ***All^a^***, when taking all the *Pinus massoniana* Lamb seedling under the three management as a whole. Data shown are the mean ± standard deviation (n = 3). Different lowercase letters indicated significant differences among the treatments (*p* < 0.05).

**Supplementary Table 2**

The mean (Mean), minimum (Min), and maximum (Max) stand spatial structure indexes

| Treatment | ***M*** | | | ***W*** | | | ***CI*** | | | ***U*** | | | ***O*** | | |
| --- | --- | --- | --- | --- | --- | --- | --- | --- | --- | --- | --- | --- | --- | --- | --- |
|  | Mean | Min | Max | Mean | Min | Max | Mean | Min | Max | Mean | Min | Max | Mean | Min | Max |
| *LCT* | 0.1237 | 0.12 | 0.13 | 0.4133 | 0.41 | 0.42 | 135.2230 | 132.87 | 137.57 | 0.4873 | 0.47 | 0.50 | 0.3647 | 0.36 | 0.37 |
| *LIT* | 0.0960 | 0.09 | 0.10 | 0.4117 | 0.41 | 0.42 | 181.8350 | 179.04 | 184.63 | 0.5040 | 0.50 | 0.51 | 0.2757 | 0.26 | 0.29 |
| *CK* | 0.0907 | 0.08 | 0.10 | 0.4017 | 0.40 | 0.40 | 235.9410 | 232.38 | 239.51 | 0.5187 | 0.51 | 0.53 | 0.1783 | 0.18 | 0.18 |
| *All^a^* | 0.1034 | 0.08 | 0.13 | 0.4089 | 0.40 | 0.42 | 184.3330 | 132.87 | 239.51 | 0.5033 | 0.47 | 0.53 | 0.2729 | 0.18 | 0.37 |

**Note:** The logging methods are ***LCT***, logging of the competition trees; ***LIT***, logging of the inferior trees; ***CK***, unselected-logging; ***All^a^***, when taking all the *Pinus massoniana* Lamb seedling under the three management as a whole. The stand spatial structure parameters are ***M***, mingling index; ***W***, uniform angle index; ***CI***, competition index; ***U***, neighborhood comparison; ***O***, opening degree. **“Mean”** represents the mean *M/ W/ CI/ U/ O* of stand spatial structure indexes, **“Min”/“Max”** represents the mean *M/ W/ CI/ U/ O* of stand spatial structure indexes.

**Supplementary Table 3**

The mean (Mean), minimum (Min), and maximum (Max) environment factors under the three treatments

| Indexes | | ***LCT*** | ***LIT*** | ***CK*** | ***All^a^*** |
| --- | --- | --- | --- | --- | --- |
| **SOC/%** | Mean | 32.41 | 31.46 | 28.30 | 30.72 |
|  | Min | 31.62 | 30.37 | 25.75 | 25.75 |
|  | Max | 32.88 | 32.77 | 29.60 | 32.88 |
| **STN/g·kg^-1^** | Mean | 2.24 | 2.14 | 2.15 | 2.17 |
|  | Min | 2.17 | 2.11 | 2.13 | 2.11 |
|  | Max | 2.27 | 2.18 | 2.16 | 2.27 |
| **SAN/mg·kg^-1^** | Mean | 169.38 | 152.41 | 142.96 | 154.92 |
|  | Min | 164.84 | 151.92 | 142.52 | 142.52 |
|  | Max | 171.82 | 152.83 | 143.24 | 171.82 |
| **STP/g·kg^-1^** | Mean | 0.22 | 0.21 | 0.22 | 0.22 |
|  | Min | 0.21 | 0.21 | 0.22 | 0.21 |
|  | Max | 0.22 | 0.21 | 0.22 | 0.22 |
| **SAP/mg·kg^-1^** | Mean | 1.52 | 1.12 | 1.21 | 1.28 |
|  | Min | 1.36 | 1.12 | 1.11 | 1.11 |
|  | Max | 1.68 | 1.13 | 1.34 | 1.68 |
| **SK/g·kg^-1^** | Mean | 3.86 | 4.20 | 3.49 | 3.85 |
|  | Min | 3.57 | 3.86 | 3.17 | 3.17 |
|  | Max | 4.23 | 4.73 | 3.97 | 4.73 |
| **SAK/mg·kg^-1^** | Mean | 0.23 | 0.20 | 0.16 | 0.20 |
|  | Min | 0.22 | 0.19 | 0.15 | 0.15 |
|  | Max | 0.26 | 0.20 | 0.17 | 0.26 |
| **SW/%** | Mean | 18.65 | 17.02 | 20.75 | 18.80 |
|  | Min | 17.36 | 16.28 | 18.47 | 16.28 |
|  | Max | 20.05 | 17.64 | 22.46 | 22.46 |
| **pH** | Mean | 5.08 | 5.23 | 5.15 | 5.15 |
|  | Min | 4.67 | 5.08 | 5.02 | 4.67 |
|  | Max | 5.30 | 5.35 | 5.29 | 5.35 |
| **SBD/g·cm^-3^** | Mean | 1.43 | 1.32 | 1.33 | 1.36 |
|  | Min | 1.40 | 1.31 | 1.32 | 1.31 |
|  | Max | 1.47 | 1.32 | 1.35 | 1.47 |
| **SP/%** | Mean | 57.17 | 56.97 | 56.13 | 56.76 |
|  | Min | 56.71 | 55.64 | 55.91 | 55.91 |
|  | Max | 57.53 | 58.53 | 56.13 | 58.53 |

**Note:** The logging methods are ***LCT***, logging of the competition trees; ***LIT***, logging of the inferior trees; ***CK***, unselected-logging; ***All^a^***, when taking all the *Pinus massoniana* Lamb seedling under the three management as a whole. The soil parameters are **SOC**, soil organic carbon; **STN**, soil total nitrogen; **SAN**, soil alkaline nitrogen; **STP**, soil total phosphorus; **SAP**, soil available phosphorus; **SK**, soil total potassium; **SAK**, soil available potassium; **SW**, soil water content; **pH**, soil pH; **SBD**, soil bulk density; **SP**, soil porosity. **“Mean”** represents the mean SOC/ STN/ SAN/ STP/ SAP/ SK/ SAK/ SW/ pH/ SBD/ SP of the soil, **“Min”/“Max”** represents the mean SOC/ STN/ SAN/ STP/ SAP/ SK/ SAK/ SW/ pH/ SBD/ SP of the soil.

**Supplementary Table 4**

The mean (Mean), minimum (Min), and maximum (Max) height and ground-diameter of *Pinus massoniana Lamb* seedling under the different management treatments

| Treatment | Height/cm | | | Ground-diameter/mm | | | W_B_/g·plant^-1^ | | | W_F_/g·plant^-1^ | | | W_S_/g·plant^-1^ | | | W_R_/g·plant^-1^ | | | W_T_/g·plant^-1^ | | |
| --- | --- | --- | --- | --- | --- | --- | --- | --- | --- | --- | --- | --- | --- | --- | --- | --- | --- | --- | --- | --- | --- |
|  | Mean | Min | Max | Mean | Min | Max | Mean | Min | Max | Mean | Min | Max | Mean | Min | Max | Mean | Min | Max | Mean | Min | Max |
| *LCT* | 46.49 | 41.70 | 57.80 | 4.62 | 3.25 | 6.87 | 2.30 | 2.09 | 2.57 | 3.59 | 3.16 | 4.17 | 4.85 | 3.83 | 5.68 | 1.89 | 1.40 | 2.72 | 12.64 | 11.15 | 13.76 |
| *LIT* | 55.06 | 43.70 | 77.30 | 3.97 | 1.81 | 6.60 | 1.07 | 0.88 | 1.33 | 2.07 | 1.84 | 2.41 | 2.62 | 2.34 | 3.14 | 1.32 | 1.22 | 1.55 | 7.09 | 6.52 | 8.35 |
| *CK* | 37.82 | 23.70 | 52.32 | 2.95 | 2.16 | 3.75 | 0.69 | 0.57 | 0.97 | 1.00 | 0.77 | 1.25 | 2.33 | 1.60 | 2.73 | 0.84 | 0.53 | 1.25 | 4.86 | 3.48 | 5.76 |
| *All^a^* | 46.38 | 23.70 | 77.30 | 3.84 | 1.81 | 6.87 | 1.36 | 0.57 | 2.57 | 2.22 | 0.77 | 4.17 | 3.27 | 1.60 | 5.68 | 1.35 | 0.53 | 2.72 | 8.21 | 3.48 | 13.76 |

**Note:** The logging methods are ***LCT***, logging of the competition trees; ***LIT***, logging of the inferior trees; ***CK***, unselected-logging; ***All^a^***, when taking all the *Pinus massoniana* Lamb seedling under the three management as a whole. The growth indexes are **W_B_**, branch biomass; **W_F_**, foliage biomass; **W_S_**, stem biomass; **W_R_**, root biomass; **W_T_**, total biomass. **“Mean”** represents the mean Height/ Ground-diameter/ W_B_/ W_F_/ W_S_/ W_R_/ W_T_ of *P. massoniana* seedling, **“Min”/“Max”** represents the mean Height/ Ground-diameter/ W_B_/ W_F_/ W_S_/ W_R_/ W_T_ of *P. massoniana* seedling.

**Supplementary Table 5**

The mean (Mean), minimum (Min), and maximum (Max) biomass allocation of organs and the growth index of *Pinus massoniana Lamb* seedling under the different management treatments

| Treatment | BMF (%) | | | LMF (%) | | | SMF (%) | | | RMF (%) | | | C/R ratio | | | H/D ratio | | | SQI | | |
| --- | --- | --- | --- | --- | --- | --- | --- | --- | --- | --- | --- | --- | --- | --- | --- | --- | --- | --- | --- | --- | --- |
|  | Mean | Min | Max | Mean | Min | Max | Mean | Min | Max | Mean | Min | Max | Mean | Min | Max | Mean | Min | Max | Mean | Min | Max |
| *LCT* | 18.20 | 16.07 | 20.61 | 28.48 | 25.24 | 32.30 | 38.28 | 32.17 | 41.72 | 15.03 | 11.26 | 20.74 | 3.18 | 2.27 | 4.32 | 10.33 | 6.16 | 13.93 | 0.95 | 0.71 | 1.36 |
| *LIT* | 15.15 | 11.96 | 17.11 | 29.21 | 26.75 | 33.54 | 37.00 | 34.88 | 38.80 | 18.65 | 17.04 | 20.01 | 2.38 | 2.20 | 2.82 | 14.54 | 6.86 | 27.24 | 0.44 | 0.25 | 0.78 |
| *CK* | 14.24 | 11.71 | 18.02 | 20.63 | 17.70 | 24.23 | 47.94 | 41.83 | 54.44 | 17.19 | 14.59 | 21.65 | 2.04 | 1.69 | 2.70 | 12.95 | 8.27 | 17.60 | 0.33 | 0.24 | 0.47 |
| *All^a^* | 15.87 | 11.71 | 20.61 | 26.08 | 17.70 | 33.54 | 41.11 | 32.17 | 52.44 | 16.94 | 11.26 | 21.65 | 2.53 | 1.69 | 4.32 | 12.59 | 6.16 | 27.24 | 0.57 | 0.24 | 1.36 |

**Note:** The logging methods are ***LCT***, logging of the competition trees; ***LIT***, logging of the inferior trees; ***CK***, unselected-logging; ***All^a^***, when taking all the *Pinus massoniana* Lamb seedling under the three management as a whole. The growth indexes are **BMF**, branch mass fraction; **LMF**, foliage mass fraction; **SMF**, stem mass fraction; **RMF**, root mass fraction; **C/R ratio**, crown-root mass fraction ratio; **H/D ratio**, height-ground-diameter ratio; **SQI**, seedling quality index. **“Mean”** represents the mean BMF/ LMF/ SMF/ RMF/ C-R ratio/ H-D ratio/ SQI of *P. massoniana* seedling, **“Min”/“Max”** represents the mean BMF/ LMF/ SMF/ RMF/ C-R ratio/ H-D ratio/ SQI of *P. massoniana* seedling.
